# Genome-wide protein–DNA interaction site mapping in bacteria using a double-stranded DNA-specific cytosine deaminase

**DOI:** 10.1038/s41564-022-01133-9

**Published:** 2022-06-01

**Authors:** Larry A. Gallagher, Elena Velazquez, S. Brook Peterson, James C. Charity, Matthew C. Radey, Michael J. Gebhardt, FoSheng Hsu, Lauren M. Shull, Kevin J. Cutler, Keven Macareno, Marcos H. de Moraes, Kelsi M. Penewit, Jennifer Kim, Pia A. Andrade, Thomas LaFramboise, Stephen J. Salipante, Michelle L. Reniere, Victor de Lorenzo, Paul A. Wiggins, Simon L. Dove, Joseph D. Mougous

**Affiliations:** 1 Department of Microbiology, University of Washington, Seattle, WA USA; 2 Systems Biology Department, National Center of Biotechnology CSIC, Madrid, Spain; 3 Division of Infectious Diseases, Boston Children’s Hospital, Harvard Medical School, Boston, MA USA; 4 Department of Physics, University of Washington, Seattle, WA USA; 5 Department of Laboratory Medicine and Pathology, University of Washington, Seattle, WA USA; 6 Department of Genetics and Genome Sciences, Case Western Reserve University, Cleveland, OH USA; 7 Department of Bioengineering, University of Washington, Seattle, WA USA; 8 Department of Biochemistry, University of Washington School of Medicine, Seattle, WA USA; 9 Howard Hughes Medical Institute, University of Washington, Seattle, WA USA

**Keywords:** Microbiology, Applied microbiology

## Abstract

DNA–protein interactions are central to fundamental cellular processes, yet widely implemented technologies for measuring these interactions on a genome scale in bacteria are laborious and capture only a snapshot of binding events. We devised a facile method for mapping DNA–protein interaction sites in vivo using the double-stranded DNA-specific cytosine deaminase toxin DddA. In 3D-seq (DddA-sequencing), strains containing DddA fused to a DNA-binding protein of interest accumulate characteristic mutations in DNA sequence adjacent to sites occupied by the DNA-bound fusion protein. High-depth sequencing enables detection of sites of increased mutation frequency in these strains, yielding genome-wide maps of DNA–protein interaction sites. We validated 3D-seq for four transcription regulators in two bacterial species, *Pseudomonas aeruginosa* and *Escherichia coli*. We show that 3D-seq offers ease of implementation, the ability to record binding event signatures over time and the capacity for single-cell resolution.

## Abstract (web summary)

A method that exploits the double-stranded DNA-targeting capability of the bacterial toxin-derived cytosine deaminase, DddA, can map DNA–protein interactions in bacteria in vivo at genome-wide scale.

## Main

Advances in DNA sequencing have promoted rapid expansion in DNA–protein interaction (DPI) mapping technologies. Among these, chromatin immunoprecipitation sequencing (ChIP-seq) is a well-established method for studying DPIs in both prokaryotic and eukaryotic systems^1^. In this approach, DPIs are identified by chemical crosslinking of DNA–protein complexes, DNA fragmentation, immunoprecipitation of a DNA-binding protein (DBP) of interest, crosslink reversal, DNA purification and DNA sequencing. More recently, Cut&Run and related technologies have gained popularity as alternatives to ChIP-seq^2,3^. These techniques offer several advantages including small quantities of starting material that permit single-cell measurements, the absence of crosslinking and its associated artefacts, and reduced sequencing with improved signal-to-noise^4–6^.

Although powerful, ChIP-seq and related approaches are endpoint measurement-based technologies and cannot record DPIs in living cells. One method that addresses this limitation is DNA adenine methyltransferase identification (DamID), in which the DNA-binding protein of interest is fused to DAM and DPI site identification occurs through restriction enzyme or antibody mediated methylation site enrichment^7^. However, the utility of this technique is limited by low resolution (1 kb) owing to the frequency of DAM recognition sites (GATC) and by toxicity resulting from widespread adenine methylation. A second approach that maps DPIs in living cells employs self-reporting transposons (SRTs). In this technique, a transposase is fused to the DBP of interest, and DPIs are identified by DNA or RNA sequencing to determine sites of transposon insertion^8,9^. A limitation of this approach is that transposon insertions occur at low frequency within individual cells (15–100 events per cell), so this method is not amenable to single-cell studies^8^. Additionally, the accumulation of transposon insertions within a population may cause phenotypic consequences through gene disruption.

Nucleic acid-targeting deaminases are a diverse group of proteins that have found a number of biotechnological applications due to their ability to introduce mutations in DNA or RNA. Fusion of the single-stranded DNA (ssDNA) cytosine deaminase APOBEC to catalytically inactive or nickase variants of Cas9 led to the development of the first precision base editor capable of introducing single nucleotide substitutions (C•G-to-T•A) in vivo^10^. This breakthrough technology inspired the repurposing of several other ssDNA and RNA-targeting deaminases as base editing tools, including editors that catalyze A•T-to-G•C substitutions in DNA, and RNA transcript editors that induce C to U or A to I modifications^11^. RNA-targeting deaminases have additionally been employed for the identification of RNA–protein complex sites^12^. As the only deaminase known to act preferentially on double-stranded DNA, the bacterial toxin-derived cytosine deaminase, DddA, is unique. We previously capitalized on this feature to develop DddA-derived cytosine base editors (DdCBEs) composed of DddA–TALE (transcription activator-like effector) fusions that edit the human mitochondrial genome in a programmable fashion^13^.

Here we harnessed the dsDNA-targeting capability of DddA to develop 3D-seq, a technique for genome-wide DPI mapping within bacteria. Since this method utilizes fixed mutational signatures catalyzed by the deaminase to identify DPIs, it offers the ability to study DPIs removed temporally from their occurrence and permits the analysis of DPIs in single cells.

## Results

### 3D-seq for in vivo DNA–protein interaction mapping in bacteria

**Fig. 1:**
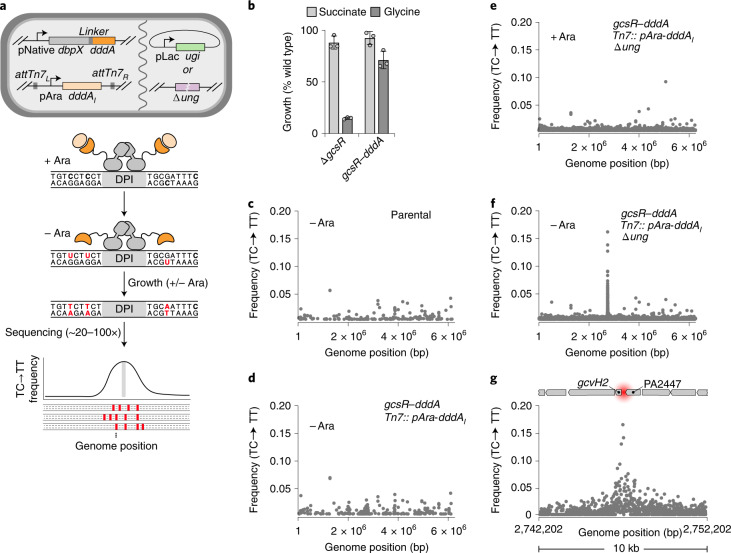
3D-seq for in vivo DNA–protein interaction mapping in *P. aeruginosa*. **a**, Diagram providing an overview of the 3D-seq method. Top: cell schematic containing the genetic elements required for 3D-seq. Elements may be integrated into the chromosome or supplied on plasmids. Middle: model depicting localized activity of DddA (dark orange) when fused to a DBP of interest (grey) and after growth in the absence of arabinose to limit production of DddA_I_ (light orange). Bottom: schematized 3D-seq output indicating enrichment of C•G-to-T•A transitions (red) in the vicinity of a DPI site (grey). **b**, Growth yield (normalized to WT) of the indicated strains on minimal medium containing glycine or succinate as the sole carbon source. Mean ± s.d. are shown; *n* = 3 biologically independent cultures, and results are representative of two experiments conducted. **c**–**f**, Average (*n* = 4) C•G-to-T•A transition frequency by genome position after passaging cultures of *P. aeruginosa* bearing the indicated genotypes, in the presence or absence of arabinose (Ara) to induce DddA_I_ expression. Data were filtered to remove a prophage hypervariable region and positions with low sequence coverage (<15-fold read depth). **g**, Zoomed view of a subset of the data depicted in **f**. Approximate location of the previously characterized GcsR binding sites (red) and adjacent genetic elements are shown to scale at the top. Source data

In DdCBEs, DddA activity is localized to particular sites on DNA by reconstitution of the enzymatic domain of the toxin (amino acids 1,264–1,427) from split forms fused to sequence-specific targeting proteins^13^. We envisioned an inverse approach whereby fusion of the intact deaminase domain of DddA, referred to herein as DddA, to DBPs with unknown binding sites could be used to define sites of interaction (Fig. 1a). To test the feasibility of this approach, we selected the DBP GcsR of *P. aeruginosa*. GcsR is a σ^54^-dependent transcription activator of an operon encoding the glycine cleavage system (*gcvH2*, *gcvP2* and *gcvT2*) and auxiliary glycine and serine metabolic genes (*glyA2* and *sdaA*)^14^. By analogy with closely related σ^54^-dependent regulators, also referred to as bacterial enhancer binding proteins (bEBPs), glycine binding to GcsR is thought to activate transcription of the operon by triggering conformational changes among subunits bound to three 18 bp tandem repeat sequences in the *gcvH2* promoter region. RNA-seq analyses of *P. aeruginosa* ∆*gcsR* suggest that the *gcvH2* operon may encompass the only genes subject to direct regulation by GcsR^14^.

To capture physiologically relevant DNA binding, we sought to generate a GcsR–DddA translational fusion encoded at the native *gcsR* locus. These efforts revealed that even in the context of fusion to transcription factors under native regulation, DddA exhibits sufficient toxicity to interfere with strain construction. To circumvent this, we inserted the gene encoding the DddA cognate immunity determinant, *dddA*_*I*_, at the Tn7 attachment site under the control of an arabinose-inducible promoter (pAra). In this background, and with induction of immunity, we successfully replaced *gcsR* with an open reading frame encoding GcsR bearing an unstructured linker at its C terminus fused to the deaminase domain of DddA (GcsR–DddA). Activation of the *gcvH2* operon by GcsR is required for *P. aeruginosa* growth using glycine as a sole carbon source^14^. Unlike a strain lacking GcsR, strains expressing GcsR–DddA utilize glycine as a growth substrate, suggesting that the fusion retains functionality (Fig. 1b).

**Extended Data Fig. 1:**
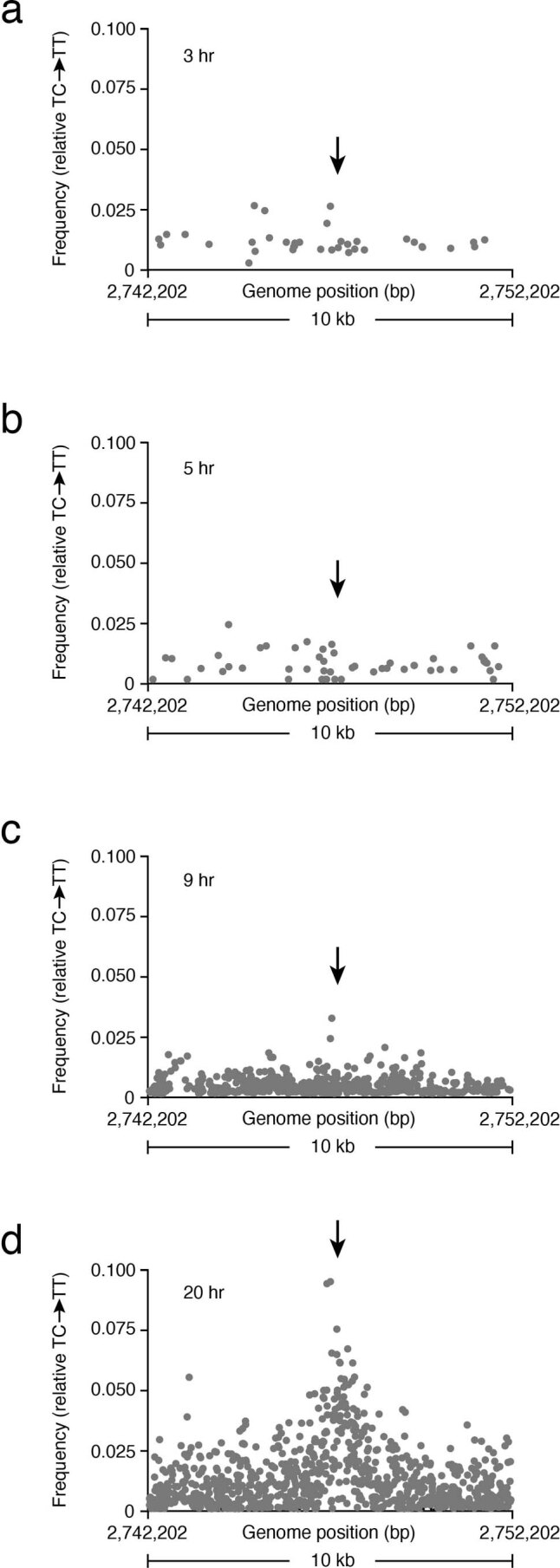
Transition mutations associated with GcsR:DddA activity accumulate over time. **a-d**, Average (n = 4) C•G-to-T•A transition frequency within the primary GcsR 3D-seq peak region after the indicated growth period and in the absence of arabinose. Data were filtered as in Fig. 1. The arrow indicates the approximate position of the known GcsR binding site. Source data

Uracil DNA glycosylase (Ung) effectively inhibits uracil accumulation in cells exposed to DddA^15^. Reasoning that this DNA repair factor would limit our capacity to detect DddA activity, we deleted *ung* in the GcsR–DddA-expressing strain. Next we passaged this strain in the presence and absence of arabinose and performed Illumina-based whole-genome sequencing. Data from replicate experiments were filtered to remove positions with low coverage or hypervariability (Methods) and the average frequency of C•G-to-T•A transition events within 5´-TC-3´ contexts were visualized across the *P. aeruginosa* genome (Fig. 1c–f). Other dinucleotide contexts were excluded on the basis of the known strong preference of DddA for thymidine at the −1 position^13^. Remarkably, in samples propagated in the absence of arabinose, we observed a single apparent peak of DddA activity, which was localized to the promoter region of *gcvH2* (Fig. 1f,g). This peak was not observed in samples containing arabinose, nor was it present in parallel studies using a strain containing Ung (Fig. 1d,e). Further studies showed that DddA activity at the *gcvH2* promoter could be detected as early as 9 h following removal of immunity inducer (Extended Data Fig. 1).

**Fig. 2:**
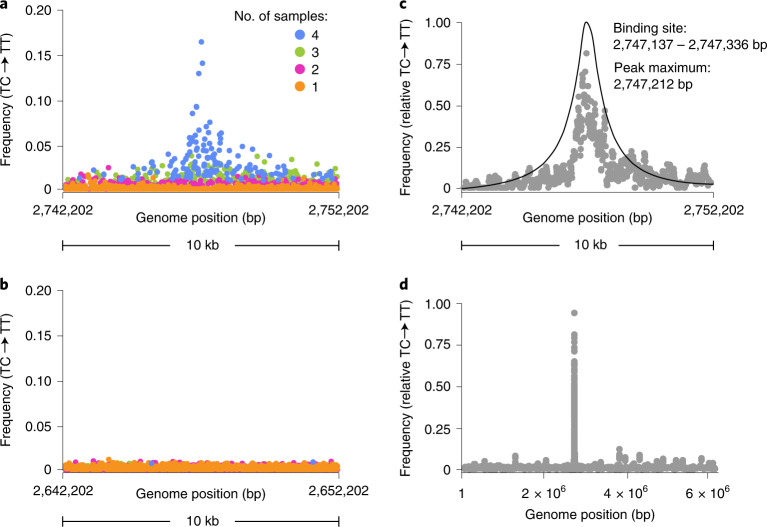
Statistical analyses and data filtering for enhanced 3D-seq precision. **a**,**b**, Average (*n* = 4) C•G-to-T•A transition frequency within the primary GcsR 3D-seq peak region (**a**) or a control region located 100,000 bp upstream (**b**), with positions coloured by the number of replicates in which a transition at that position was observed. **c**, Moving average (75 bp window) of C•G-to-T•A transition frequencies and the curve derived from our statistical model (black line) calculated from filtered 3D-seq data for the GcsR peak region (Methods). *Y*-coordinates for the model curve are scaled arbitrarily. **d**, Genome-wide moving average (75 bp window) of C•G-to-T•A transition frequencies calculated for GcsR 3D-seq data after filtering as in **c**. Source data

While a single peak of GcsR–DddA-dependent activity was readily apparent in our minimally processed data, we reasoned that additional filtering to remove background signal would improve the sensitivity and accuracy of our technique. Given our previous observation that modifications catalyzed by free DddA are randomly distributed across genomes, we reasoned that substantial noise reduction could be achieved by removing transitions not reproduced in independent replicates. Visualization of four GcsR–DddA replicate datasets showed that transition events observed in at least three of the samples were highly enriched in the peak region associated with the *gcvH2* promoter (Fig. 2a,b), and therefore this criterion was added to our filtering workflow.

In parallel, we sought to develop a statistical analysis able to provide a quantitative means of distinguishing specific DPIs from the background within 3D-seq data. Our approach employed a null hypothesis test and is described in detail in Supplementary Note 1. Briefly, a null hypothesis consisting of only background enzyme activity was compared to an alternative hypothesis in which a single putative peak was fit by maximum likelihood analysis. The null hypothesis was then either accepted or rejected at a confidence level of 95% using a Generalized Likelihood Ratio test. If the null hypothesis was rejected, the model containing the peak replaced the null hypothesis and the test was repeated for another putative peak until no more peaks could be detected. *P* values for each peak detected in this study are reported in Supplementary Table 1. The application of these filtering criteria and statistical analyses to our GcsR 3D-seq data dramatically improved signal-to-noise and placed the major GcsR–DddA binding site centred within the 200 bp region containing the three known binding sites for GcsR^14^ (Fig. 2c,d).

**Extended Data Fig. 2:**
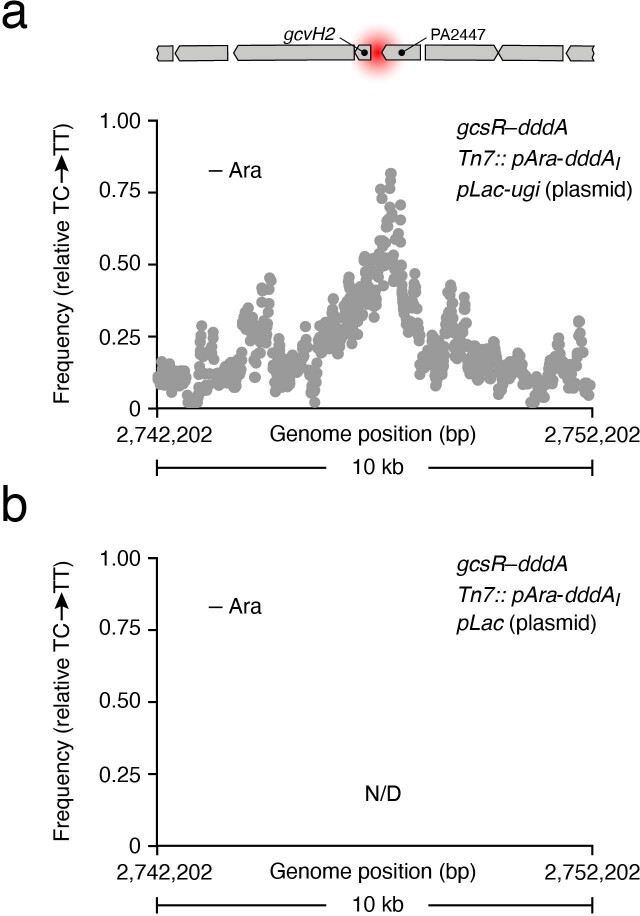
Ugi expression can substitute for genetic inactivation of *ung* in 3D-seq. **a**, **b**, Moving average (n = 4, 75 bp window) of C•G-to-T•A transition frequencies calculated from filtered 3D-seq data deriving from the indicated *P. aeruginosa* strains grown in the absence of arabinose for 20 hrs. IPTG was included to induce the expression of Ugi throughout the growth period. The location of the previously characterized GcsR binding site (red) and adjacent genetic elements are shown to scale above. Source data

Inactivation of Ung of the base excision repair (BER) pathway is critical for the detection of GcsR–DNA interactions by 3D-seq (Fig. 1d,f). As an alternative to an *ung* knockout, we considered whether expression of the Ung inhibitor protein, UGI, could achieve sufficient Ung inactivation to reveal GcsR DPIs^16^. This approach is potentially advantageous for 3D-seq in organisms that are difficult to modify genetically, and should be widely applicable given that Ung and the BER pathway are highly conserved, and UGI effectively inhibits Ung from organisms as diverse as bacteria and humans^13,17^. To determine whether expression of UGI could substitute for genetic inactivation of *ung*, we supplied *P. aeruginosa* expressing GcsR–DddA and DddA_I_ with a plasmid possessing Ugi under the control of the *lac*UV5 promoter to allow orthogonal modulation of DddA_I_ (arabinose) and Ugi (isopropyl β-d-1-thiogalactopyranoside (IPTG)). As when Ung was inactivated genetically, we found that inhibition of Ung by UGI expression yielded a highly significant peak of C•G-to-T•A transition events centred on the known GcsR binding site upstream of *gcvH2* (Extended Data Fig. 2a and Supplementary Table 1). This peak was not observed in the empty vector control strain (Extended Data Fig. 2b). Although additional, less prominent GcsR peaks were identified in this experiment (Supplementary Table 1), several of these are in close proximity to *gcvH2* and may not reflect distinct binding sites. We speculate that the remainder of the peaks are false positives as they do not correspond to GcsR peaks identified using either 3D-seq in the ∆*ung* mutant background or using ChIP-seq. These experiments demonstrate that 3D-seq can be used to identify DPIs in cells containing native Ung.

### 3D-seq applied to regulators with assorted features

To begin to probe the versatility of 3D-seq, we next sought to determine whether it could be used to map DPIs for a DBP that is structurally and functionally divergent from GcsR. For this analysis, we selected GacA, which belongs to a large group of transcription factors known as response regulators. Canonically, phosphorylation of these proteins by histidine kinases enhances their interaction with promoter elements, leading to modulation of transcription^18^. In the case of GacA, phosphorylation by the sensor kinase GacS promotes binding of GacA to the promoter regions of two small RNA genes, *rsmY* and *rsmZ*^19^. GacS is itself regulated by a second sensor kinase, RetS, which strongly inhibits GacS phosphotransfer to GacA^20^. To further evaluate the capacity of 3D-seq to capture the effects of posttranslational regulation of a transcription factor, we performed our studies in both Δ*gacS* and Δ*retS* backgrounds of *P. aeruginosa*.

**Extended Data Fig. 3:**
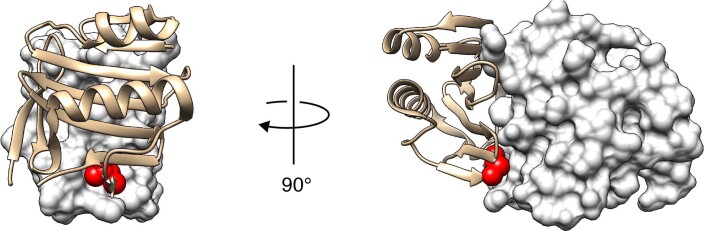
The C-terminus of DddA_I_ abuts DddA. X-ray crystal structure of the DddA_I_–DddA complex in ribbon and surface representation, respectively. The C-terminal amino acid of DddA_I_ (Leu123) is colored red and shown in space filling representation to highlight its position against the surface of DddA. The figure highlights how a C-terminal epitope fusion to DddA_I_ would weaken the interaction with DddA due to steric clash.

During preliminary 3D-seq studies with GacA, we found that repressing DddA_I_ production by removing arabinose did not lead to detectable DddA activity. We reasoned that leaky expression of DddA_I_ or variability in the accessibility of DddA in the context of different DBP–DddA fusions might produce this effect. Fortuitously, we had generated a DddA_I_ variant in which its interaction with DddA is probably weakened by a C-terminal FLAG epitope fusion (DddA_I_–F, Extended Data Fig. 3). At high arabinose levels, DddA_I_–F provided sufficient protection against DddA to permit strain construction and under lower arabinose levels, DddA-dependent C•G-to-T•A transitions were observed.

**Fig. 3:**
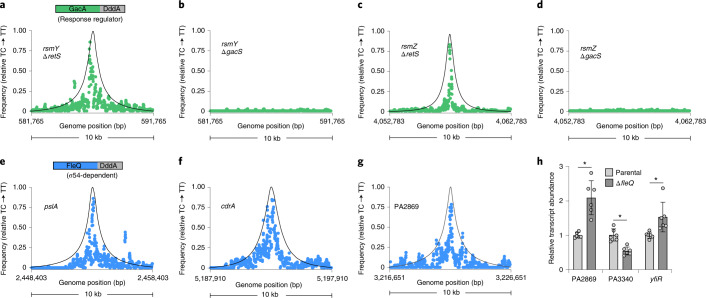
3D-seq maps DNA–protein interactions for *P. aeruginosa* transcription factors with multiple binding sites. **a**–**g**, Moving average (*n* = 4, 75 bp window) of C•G-to-T•A transition frequencies at genomic locations encompassing known binding sites upstream of the indicated genes for GacA and FleQ. Points calculated from filtered 3D-seq data derived from *P. aeruginosa* strains expressing GacA–DddA (**a**–**d**) or FleQ–DddA (**e**–**g**) grown with 0.0005% w/v arabinose for induction of DddA_I_–F. The genetic background of strains employed are noted where applicable (**a**–**d**). **h**, Abundance of transcripts encoding the indicated genes downstream of FleQ DPI sites discovered herein by 3D-seq. Mean ± s.d. are shown; *n* = 6 biologically independent samples. *P < 0.05, two-tailed paired *t*-test comparing Δ*fleQ* and WT expression for each gene (PA2869, *P* = 0.0022; PA3340, *P* = 0.0015; *yfiR*, *P* = 0.035). Source data

Consistent with previous studies, 3D-seq revealed GacA binding sites upstream of *rsmY* and *rsmZ* in the ∆*retS* background of *P. aeruginosa* (Fig. 3a,c and Supplementary Table 1). These peaks were the only significant GacA binding sites detected and were not found in the ∆*gacS* strain (Fig. 3b,d and Supplementary Table 1). These results further demonstrate the utility of 3D-seq for DPI site identification. Finally, the differential 3D-seq signal derived from strains bearing active (∆*retS*) versus inactive (∆*gacS*) GacA suggests that 3D-seq could be used to assess the activation state of regulatory proteins within bacterial populations.

Although they represent different transcription factor families, our findings show that GcsR and GacA both interact with a limited number of sites on the *P. aeruginosa* chromosome. To gauge the performance of 3D-seq with a DBP with many predicted sites of interaction, we selected FleQ. This protein is an unusual member of the bEBP family, as it can act as both an activator and repressor, it regulates transcription from both σ^54^- and σ^70^-dependent promoters, and its regulatory functions appear to be modulated by interaction with an additional protein that does not bind DNA directly^21–24^. In its capacity as a σ^54^-dependent transcription activator, in vitro studies have shown that FleQ binds the promoters of several flagellar gene operons; as a σ^70^-dependent regulator, it interacts with binding sites adjacent to or overlapping with transcription start sites for several genes involved in exopolysaccharide biosynthesis and can serve as both a repressor and an activator, depending on availability of the second messenger cyclic-di-GMP^21,23,25^.

**Extended Data Fig. 4:**
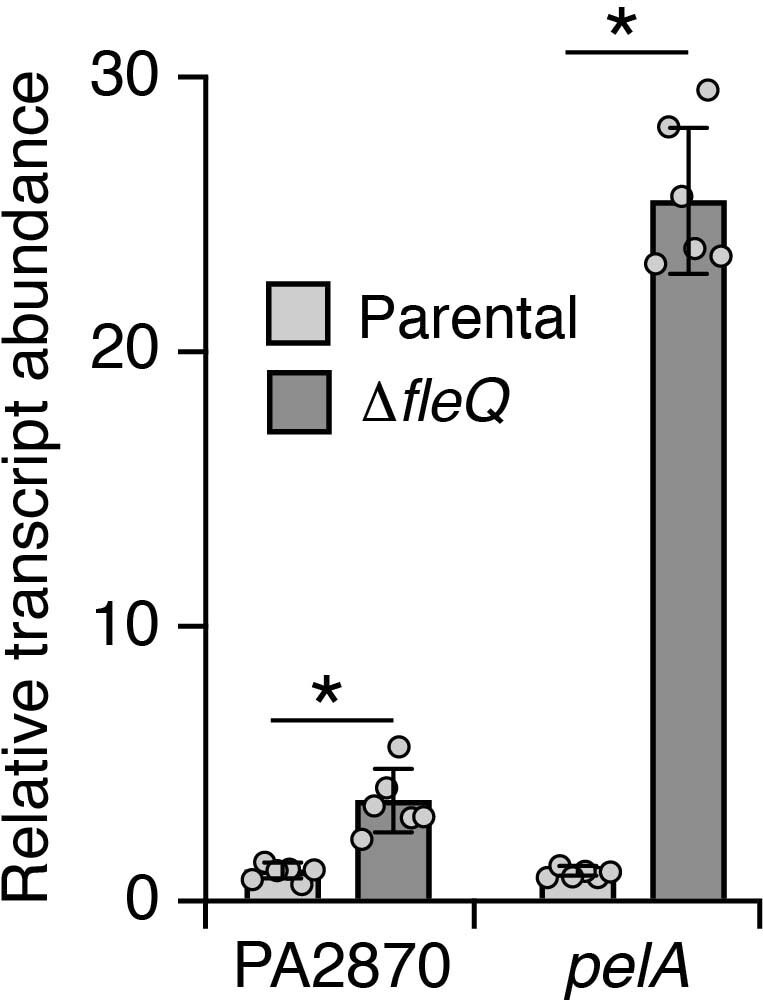
FleQ regulates a predicted cyclic-di-GMP cyclase encoded downstream of a 3D-seq-predicted binding site. Relative abundance of transcripts encoding the predicted diguanylate cyclase (PA2870) in the indicated strains of *P. aeruginosa* is compared to the known FleQ target *pelA*. Means ± SD are shown; n = 6 biologically independent samples. *p < 0.05, two-tailed paired t-test comparing Δ*fleQ* and WT expression for each gene (PA2870 p = 0.0018; *pelA*, p = 3.08 × 10^−6^). Source data

3D-seq analysis employing FleQ–DddA expressed from its native promoter identified 14 peaks with a significantly elevated frequency of C•G-to-T•A transition events (Supplementary Table 1). Many of these peaks were localized to previously identified FleQ binding sites. Consistent with studies employing purified FleQ, these included sites upstream of operons encoding Pel and Psl exopolysaccharide biosynthesis machinery and the CdrAB two-partner secreted adhesin, in addition to several flagellar motility genes known to be activated by the protein (for example, *flhF, fliL, motD*) (Fig. 3e,f)^21,23,25^. They also included the previously predicted FleQ binding sites upstream of the *siaA* and *bdlA* genes^25^. Interestingly, significant 3D-seq peaks were located upstream of several FleQ-controlled genes that were not known to be targeted directly by FleQ: the PA1462 gene and the *fleQ* gene itself^22,26^ (Supplementary Table 1). Finally, we detected FleQ binding sites upstream of genes that were not previously linked to FleQ, either by regulation or binding. These include a homologue of the motility gene *fimV* (PA3340), the PA2869 gene (positioned upstream of and in the same operon as PA2870 encoding a c-di-GMP biosynthetic enzyme) and *yfiR* (PA1121), which encodes a regulator of c-di-GMP synthesis^27^ (Fig. 3g and Supplementary Table 1). To test explicitly whether PA3340, PA2869, PA2870 and *yfiR* are controlled by FleQ, we compared expression of these genes in wild-type (WT) and Δ*fleQ* mutant cells using quantitative reverse-transcriptase PCR (qRT-PCR). This analysis demonstrated that expression of all four of these genes is impacted by FleQ inactivation; PA3340 is positively regulated by FleQ, while PA2689, PA2670 and *yfiR* are negatively regulated (Fig. 3h and Extended Data Fig. 4). Our 3D-seq analyses thus resulted in the identification of new FleQ-regulated genes, two of which (PA2870 and *yfiR*) may modulate the activity of FleQ through their effects on the intracellular concentration of c-di-GMP.

### Benchmarking of 3D-seq

ChIP-seq is the most commonly employed method for genome-wide DPI mapping in bacteria. We therefore sought to compare our 3D-seq results for each of the *P. aeruginosa* regulators analyzed in our study to those obtained by ChIP-seq. We opted to perform our own ChIP-seq study for each regulator given that ChIP-seq studies have not been published for GcsR, and the available ChIP-seq data for GacA and FleQ do not encompass many of their known or predicted binding sites^28^. To facilitate the immunoprecipitation step of ChIP-seq and to maintain consistency across the two methods, we inserted a VSV-G epitope tag in place of the DddA fusion at the native chromosomal locus of each regulator.

**Fig. 4:**
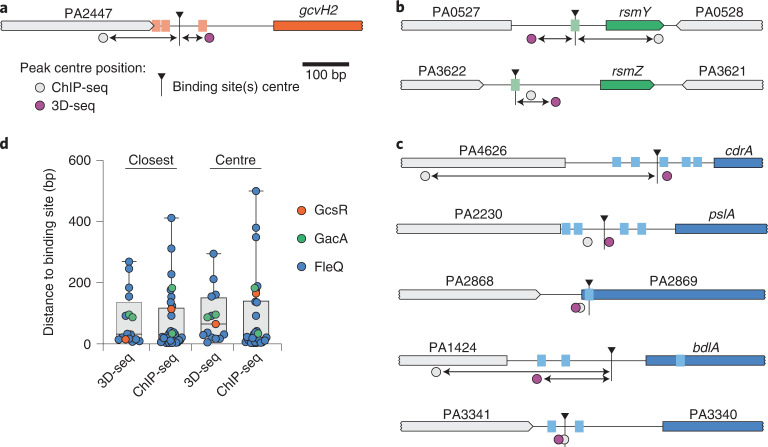
Benchmarking 3D-seq with ChIP-seq. **a**–**c**, Schematics depicting the *P. aeruginosa* genome locations of established binding motifs for GcsR (**a**), GacA (**b**) and a subset of FleQ sites (**c**). Positions of the corresponding 3D-seq and ChIP-seq peak centres defined in this study are mapped onto the regions, with extended distances to the binding site(s) centre highlighted (arrows). Colors of regulated genes and targeted sites indicate the associated regulator (orange, GcsR; green, GacA; blue, FleQ) **d**, Average distance between peaks detected by 3D-seq and ChIP-seq to the centre of the closest binding motif (left) or the midpoint of the region encompassed by multiple binding motifs (Centre, right) for the indicated regulators. *n* = 16 peaks detected by 3d-seq and 32 detected by ChIP-seq; boxes indicate median (centre) and 25th to 75th percentiles, whiskers indicate range. Source data

We began our comparison of 3D-seq and ChIP-seq by evaluating the relative sensitivity, or the number of known binding sites, detected by the two methods. GcsR and GacA are reported to bind a limited number of sites, and these have been characterized in detail through mutagenesis, electrophoretic mobility shift assays (for GcsR) and transcriptional reporter assays (GacA)^14,29,30^. For these regulators, the most significant peaks detected by both methods were localized in proximity to the previously identified binding sites (Fig. 4a,b, and Supplementary Tables 1 and 2). For GacA, its two known binding sites located upstream of *rsmY* and *rsmZ* represented the only statistically supported peaks detected by both methods, whereas both methods predicted one or more GcsR binding sites beyond its previously identified site of interaction uptream of *gcvH2*. These latter sites were relatively poorly supported statistically, non-overlapping and may represent false positive signal (Supplementary Tables 1 and 2). DNase I footprinting assays identified a FleQ binding motif present in the promoter region of genes in its regulon^25^. This 14 bp motif was identified by the motif discovery algorithm MEME in all but one of the 14 FleQ binding sites identified by 3D-seq, often in multiple adjacent copies and at intragenic locations, consistent with other σ^54^-dependent regulators (Fig. 4c)^14,31–33^. Our ChIP-seq study of FleQ yielded 40 statistically supported sites of enrichment. Notably, each of the FleQ binding sites identified by 3D-seq are encompassed in the ChIP-seq set and 12 of these are among the 20 most enriched sites we found by ChIP-seq (Supplementary Table 2). Of the 40 ChIP-seq sites we identified, 29 contained one or more FleQ binding motifs (Fig. 4c). Altogether, we conclude that 3D-seq exhibits sensitivity on par with ChIP-seq for regulators with few binding sites; however, 3D-seq can be less sensitive than ChIP-seq when examining regulators with many binding sites. Since each of the 3D-seq-identified FleQ binding sites are encompassed in the set identified by ChIP-seq and >90% of these contain the FleQ binding motif (compared with 73% for ChIP-seq), it is possible that 3D-seq data may in some cases yield fewer false positive predictions.

Next we evaluated the relative accuracy of 3D-seq and ChIP-seq by measuring the distance of their respective peak centres to the established binding sites for all three regulators. Our analysis revealed that the accuracy of the two methods is remarkably similar, irrespective of whether distance was calculated to the closest binding motif (3D-seq, 81 bp; ChIP-seq, 73 bp) or to the centre position of the region spanned by all motifs at a given promoter (3D-seq, 87 bp; ChIP-seq, 86 bp) (Fig. 4d). Thus, 3D-seq and ChIP-seq broadly appear equally capable of identifying and accurately mapping DPIs on a genome-wide scale. While ChIP-seq is a highly optimized technology, there are probably substantial optimizations that can be applied to 3D-seq to improve its performance beyond this first-generation implementation.

### 3D-seq applied to a second bacterial species

An important question regarding the future utility of 3D-seq is whether the technique is generalizable to other bacterial species. To address this, we asked whether 3D-seq could identify DPIs involving NtrC, a well-studied σ^54^-dependent transcription activator of the nitrogen stress response in *Escherichia coli*^34,35^. First, we constructed a strain of *E. coli* containing an in-frame deletion of the *ung* gene and expressing DddA_I_–F under the control of the *tac* promoter from a pMMB-derived plasmid^36^. This strain of *E. coli* was then modified further to encode NtrC–DddA at the *ntrC* native chromosomal location.

**Fig. 5:**
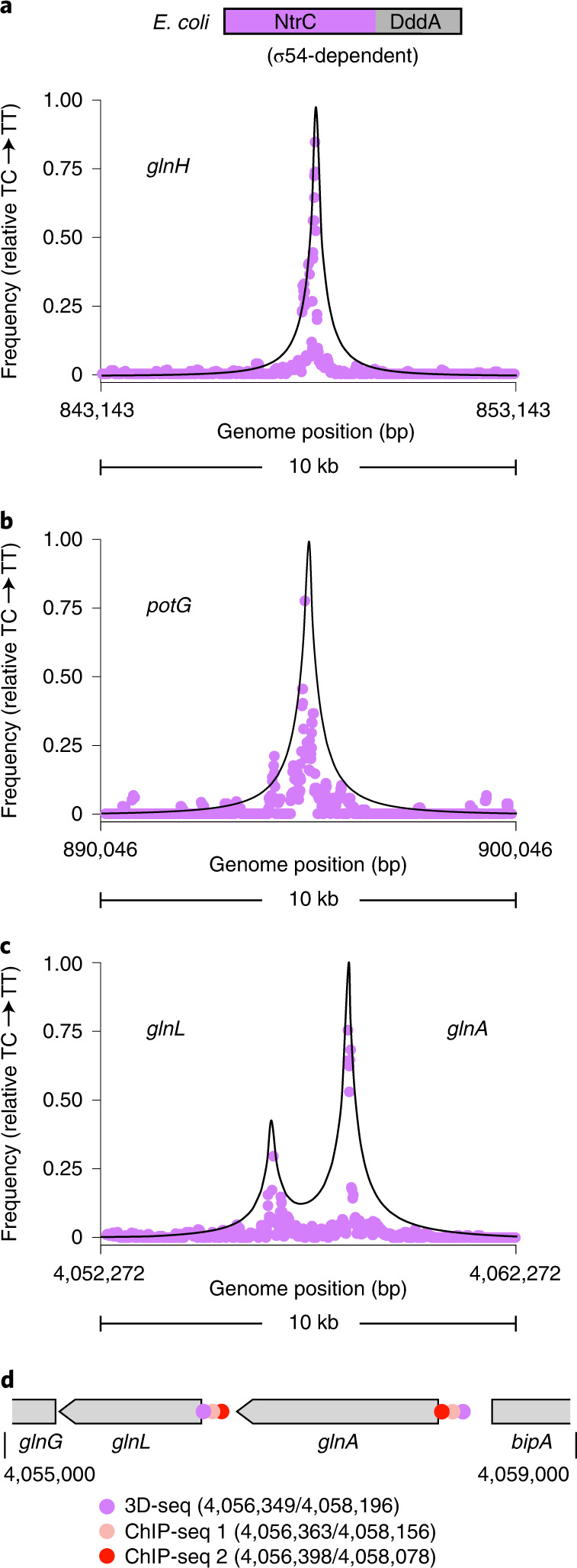
3D-seq maps binding sites of NtrC in *E. coli* with single-gene resolution. **a**–**c**, Moving average (*n* = 4, 75 bp window) of C•G-to-T•A transition frequencies at genomic locations encompassing previously predicted NtrC binding sites upstream of the indicated genes. Points were calculated from filtered 3D-seq data derived from *E. coli* expressing NtrC–DddA grown for multiple passages in minimal media with limiting nitrogen (2 mM NH_4_Cl) in the absence of IPTG for induction of DddA_I_–F. **d**, Schematic depicting the location of NtrC binding sites predicted by 3D-seq and two previous ChIP-seq studies in the vicinity of two adjacent NtrC-regulated genes, *glnA* and *glnH* (ChIP-seq 1, ref. ^34^; ChIP-seq 2, ref. ^37^). Source data

3D-seq with NtrC–DddA in cells experiencing nitrogen limitation (conditions under which NtrC is activated^34,35^) identified eight statistically significant peaks (Fig. 5a–c and Supplementary Table 1). The locations of the NtrC binding sites identified by 3D-seq agree well with those identified in two published ChIP-seq studies: one performed with epitope-tagged NtrC expressed from its native chromosomal location^34^ and one in which epitope-tagged NtrC was ectopically expressed^37^. All 3D-seq NtrC binding sites are encompassed within the ten most strongly supported sites found by each of the previous analyses. Six of the eight peaks we detected are positioned <200 bp from peaks found in both previous studies (82 bp average), while the remaining two localize <200 bp from binding sites found only in the ectopic expression study. Notably, the sites detected by 3D-seq include two found upstream of adjacent genes *glnA* and *glnH* (Fig. 5c,d). The capacity of 3D-seq to distinguish these as distinct binding sites indicates that the technique can define DPIs with single-gene resolution (Fig. 5c). Consistent with our comparison of 3D-seq and ChIP-seq results for FleQ in *P. aeruginosa*, 3D-seq identified fewer DPIs for NtrC than did ChIP-seq. However, examination of peaks detected by our algorithm that failed to meet the significance cut-off we imposed revealed predicted DPIs in close proximity (74 bp average) to six additional sites identified by ChIP-seq (Supplementary Table 1). This finding suggests that increasing sequencing depth or further exposure of cells to DBP–DddA fusions probably enhances the sensitivity of 3D-seq.

**Extended Data Fig. 5:**
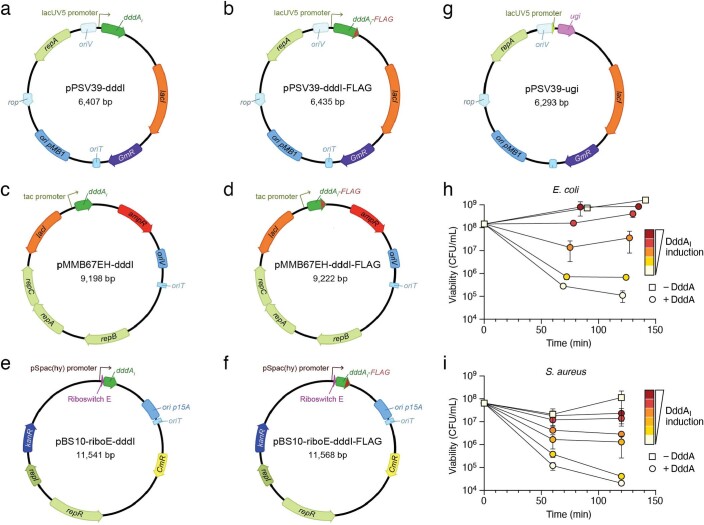
Plasmids tools to facilitate 3D-seq experiments in bacteria. Schematics of plasmids for modulated expression of DddA_I_, DddA_I_-FLAG and Ugi. **a-d**, Gram-negative broad host range plasmids pPSV39-dddI(-FLAG) and pMMB67EH-dddI(−FLAG) confer gentamycin and ampicillin/carbenicillin resistance, respectively, and can be mobilized using conjugative IncP helper plasmids (for example, pRK2013)^36,66^. DddA_I_ expression from these plasmids is inducible by IPTG from the *lacUV5* and *tac* promoters, respectively. **e,f**, Plasmids pBS10-riboE-dddI and pBS10-riboE-dddI-FLAG for regulated immunity expression in Firmicutes. A riboswitch in the constitutively expressed transcript translationally controls protein expression in response to the inducer theophylline. **g**, Plasmid for IPTG-inducible Ugi expression to inhibit uracil-N-glycosylase activity as an alternative to inactivating the bacterial *ung* gene (Supplementary Fig 2). **h**, **i**, Growth of *E. coli* (**h**) or *S. aureus* (**i**) bearing plasmids for inducible expression of DddA and DddA_I_ (pPSV39-*dddI* or pBS10-riboE-*dddI*) were grown to mid-log (**h**) or late-log (**i**) phase without inducers, then IPTG (**h**) or theophylline (**i**) was added at a range of concentrations (IPTG, 0.01, 0.022, 0.046 and 1 mM; theophylline, 0.1, 0.25, 0.5, 1, and 2 mM) to induce immunity gene expression. DddA expression was induced twenty minutes later (**h**) or concurrently (**i**) by the addition of rhamnose (**h**) or xylose (**i**), and viable titers were tracked over time. Means ± SD are shown; n = 2 biologically independent samples for *E. coli* (**h**) and 3 for *S. aureus* (**i**). Source data

The successful extension of 3D-seq to *E. coli* motivated us to generate a collection of genetic tools to aid researchers in implementing 3D-seq. Briefly, we constructed, tested and will make available for distribution a set of broad host range plasmids that express DddA_I_, DddA_I_–F and Ugi under the control of inducible promoters compatible with a wide range of organisms (Extended Data Fig. 5). We have also made our data processing and analysis scripts available on Github.

### Single-cell measurements using 3D-seq

**Fig. 6:**
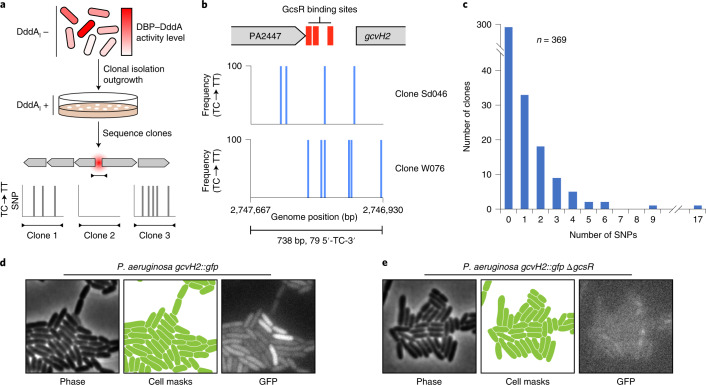
Single cell DNA–protein interaction measurements using 3D-seq. **a**, Schematic depicting the generalized workflow of a single-cell 3D-seq experiment. In the initial population, there are varying levels of activity of the DBP–DddA fusion (top), which are measured as the total number of SNPs at a target site (red sphere) following sequencing of individual clones (bottom). **b**, Single-cell 3D-seq data for *P. aeruginosa* GcsR. Locations of SNPs (blue bars) within the *gcvH2* promoter region are shown for the two indicated clones. Adjacent SNPs are not differentiated with separate bars. Clone labels correspond to those in Supplementary Table 3. **c**, Summary of SNPs detected within the sequencing window indicated in **b** among 369 *P. aeruginosa* GcsR single-cell 3D-seq clones. **d**,**e**, Cropped micrographs and corresponding computed cell mask regions for the indicated *P. aeruginosa* strains expressing green fluorescent protein (GFP) under the control of the *gcvH2* promoter. Scale bar, 1 μm. Images are representative of those collected in two independent experiments. Source data

The C•G-to-T•A transition frequency we measure to generate DPI predictions is derived from a diverse, non-clonal pool of cells. At any given parental 5´-TC-3´ site, a fraction of these bear the 5´-TT-3´ single nucleotide polymorphism (SNP) and the remaining cells are WT at the position. These data allow DPI site identification at the population level; however, they necessarily obscure information pertaining to the heterogeneity of DPIs that may exist at the single-cell level. We postulated that restoring DddA_I_ expression to this mixed population, followed by a clonal outgrowth step and sequencing, would illuminate DBP behaviour within single cells (Fig. 6a). To test this hypothesis, we propagated our *P. aeruginosa* GcsR–DddA-expressing strain in the absence of DddA_I_ inducer to allow the accumulation of mutations within the *gcvH2* promoter region. We then obtained clonal isolates from this culture via growth on solid media containing DddA_I_ inducer. Whole-genome sequencing of 84 of these isolates revealed sporadic and clone-specific TC-to-TT SNPs within the *gcvH2* promoter (Fig. 6b and Supplementary Table 3). The fact that these mutations were clonally idiosyncratic and fully penetrant indicated that DddA_I_ modulation coupled with clonal outgrowth successfully recorded the behaviour of GcsR–DddA within single cells. To more thoroughly characterize this behaviour, we analyzed the *gcvH2* promoter region of 285 additional isolates by Sanger sequencing. In line with the low level of modification in the original mixed population, many of the clones we analyzed had no SNPs (Fig. 6c and Supplementary Table 3). However, certain clones contained many SNPs, up to 17 in one instance. If the mutation rate induced by GcsR–DddA at the *gcvH2* promoter was the same in all cells, we would expect the total number of mutations across our clones to adopt a Poisson distribution. On the contrary, the distribution of mutation frequency across the 369 clones we analyzed appeared heavy-tailed and a comparison of the distribution with that expected by Poisson confirmed this observation (K–S test, *P* = 0.02) (Fig. 6c).

**Extended Data Fig. 6:**
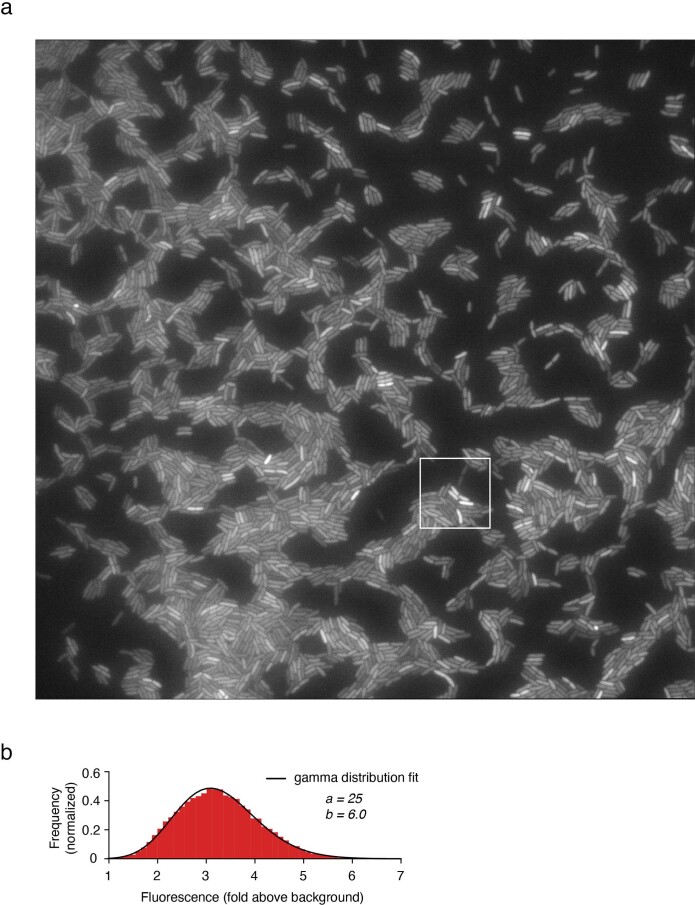
Fluorescence intensities of *P. aeruginosa* cells expressing GFP under control of the gcvH2 promoter exhibit a gamma distribution. **a**, Full field of view fluorescence micrograph of *P. aeruginosa gvh2::gfp*. Square indicates region depicted in Fig. 5d. **b**, Fluorescence intensities of >23,000 cells from the population depicted in (a). Fitted peak indicates the calculated gamma distribution. Source data

There are a number of potential explanations for the heavy-tailed distribution of SNPs observed by 3D-seq among cells expressing GcsR–DddA. We find it unlikely that this is due to DddA_I_ expression heterogeneity since arabinose was not present during the mutation accumulation stage of these experiments. To determine whether an orthogonal method would corroborate our observations, we inserted *gfp* at the *gcvH2* locus (*gcvH2*::*gfp*). Micrographs of this strain showed that cells containing this reporter exhibit a range of fluorescence intensities, with a subset of cells substantially above the mean (Fig. 6d and Extended Data Fig. 6a). However, quantification of >23,000 cells did not reveal a distinct, high-expressing population and the fluorescence intensity of all cells was well described by the gamma distribution (Extended Data Fig. 6b). A control strain lacking *gcsR* in the *gcvH2*::*gfp* background did not fluoresce above background levels, demonstrating that signal derived from *gcvH2*::*gfp* is GcsR-dependent (Fig. 6e). Together, these data suggest that the clones in which our GcsR–DddA single-cell 3D-seq analysis identified mutations probably correspond to those at the high end of the *gcvH2* expression profile, rather than an ‘on’ subpopulation. It is also possible that our 3D-seq results reflect dynamics of GcsR binding at the *gcvH2* promoter that are not captured by the indirect method of quantifying fluorescence from a translational reporter^38^. Sequencing vastly more clones (for example, using next generation-based amplicon sequencing), in conjunction with studying a population bearing higher overall modification levels, should allow 3D-seq to provide more detailed insights into DBP behaviour at the single-cell level. Nevertheless, our data clearly demonstrate that 3D-seq can be harnessed to resolve DPIs in single cells, a capability that separates the technique from other genome-wide DPI mapping techniques employed in bacteria.

## Discussion

We find that 3D-seq offers several advantages over commonly employed technologies for DPI mapping without substantial cost to accuracy or sensitivity. Key among these is its ease in implementation. The most time-intensive step in a 3D-seq assay is constructing and introducing the appropriate genetic elements. For bacteria with genetic systems in place, such as *P. aeruginosa* and *E. coli*, this process requires only 2–3 weeks. Once strain construction is complete, the 3D-seq workflow involves simply growing a small volume of the strain under examination, followed by genomic DNA preparation and whole-genome sequencing using the tagmentation library preparation method. Using this protocol, a researcher can theoretically progress from the initiation of the experiment to completed sequencing libraries within one day. Our 3D-seq protocol is also adaptable to high-throughput automated approaches. In contrast, ChIP-seq requires specialized reagents, the immunoprecipitation procedure is technically demanding and requires multiple days to complete, and sequencing library preparation for the method is more time-consuming and technically demanding than tagmentation^1^. Another distinct advantage of 3D-seq is the minimal starting material required. The lower limit on material for a 3D-seq study is defined only by the terminal DNA sequencing technology being utilized. Indeed, the genome of a single cell would be adequate for revealing DPIs by 3D-seq^39^.

There are also limitations and potential caveats to 3D-seq. Our analysis of NtrC in *E. coli* revealed that 3D-seq can distinguish binding sites with single-gene resolution. However, it is currently unable to resolve adjacent binding motifs within a promoter, probably due to their overlapping signal. Increasing sequencing depth may improve the resolution of 3D-seq, but ultimately, the resolution of 3D-seq is limited by the frequency of cytosines found in the sequence context preferred by DddA, 5´-TC-3´. In *P. aeruginosa* and *E. coli*, this dinucleotide motif occurs on average every 12 or 9 bp, respectively. Although the average frequency of 5´-TC-3´ is expected to remain relatively consistent across organisms with varying GC content, within particular genomic regions, the frequency of 5´-TC-3´ could diminish substantially and limit resolution. DddA derivatives or novel dsDNA-targeting deaminases with alternative or relaxed sequence specificity hold great promise as a solution to this limitation of 3D-seq^15,40^. Finally, the genetic manipulations required to implement 3D-seq could, in principle, have undesirable physiological effects on cells. With regard to inactivation of Ung, numerous genome editing studies in different organisms suggest that this is unlikely to have unforeseen consequences on cell physiology (for example^41–44^). The effects of mutations installed by DBP–DddA fusions are difficult to predict and will depend on the expression level and identity of the DBP. Mutations at bona fide DNA binding sites could impact expression of the regulon under investigation. Off-target mutations could also have physiological consequences but can be minimized through the timing and careful titration of DddA_I_ expression.

As performed in this study, 3D-seq exploits the small size of bacterial genomes to cost-effectively obtain high coverage (>100-fold) that can be translated into semi-quantitative measures of DBP occupancy. In eukaryotic organisms with substantially larger genomes, an approach such as this is impractical and enrichment strategies are preferable. Nevertheless, we anticipate that 3D-seq will find application in eukaryotes. If experiments are conducted in a manner that permits mutations introduced by the DBP–DddA fusion of interest to approach 100% frequency in the population, far less sequencing depth is required. In another variation, candidate sites could be amplified by PCR and amplicon sequencing would be used to reveal lower-frequency modifications.

We have generated a panel of expression plasmids for DddA_I_ that can be used to facilitate the preparation of constructs containing DddA in *E. coli*, and depending on the organism of interest and the particular regulator under study, the plasmids can further be used in 3D-seq experiments to modulate the DddA activity of a DBP–DddA fusion protein. In our experience, the optimal DddA_I_ version (DddA_I_ or DddA_I_–F) and expression level are determined empirically. Before pilot studies that use sequencing as a readout, we measure the growth characteristics of candidate 3D-seq strains expressing DddA_I_(–F) at varying induction levels. We generally find that an inducer concentration immediately below that where measurable growth inhibition occurs yields the highest signal-to-noise in 3D-seq studies.

While we have demonstrated the utility of 3D-seq for population-level and single-cell mapping of DPIs involving bacterial transcription factors under standard laboratory growth conditions, we envision that its unique features will catalyze additional applications. In particular, the ability to modulate DddA activity through DddA_I_ expression should allow 3D-seq to capture a snapshot of DPIs occurring over a fixed period of time. This could be particularly advantageous during growth under physiological conditions inaccessible to other mapping methods, such as during host colonization. As our pilot experiment employing GcsR–DddA demonstrates, the capacity to inducibly inhibit DddA also enables 3D-seq to map DPIs within single cells. Importantly, these implementations of 3D-seq are not mutually exclusive; 3D-seq could provide single cell-level resolution of DPIs within cells propagated in physiological models of interest (for example, animal colonization and infection models). In summary, we anticipate that the simplicity of 3D-seq will greatly improve the accessibility of genome-wide DPI mapping studies and its unique attributes will help usher in a new era of DPI measurements in natural contexts.

## Methods

### Bacterial strains, plasmids and growth conditions

Detailed lists of all strains and plasmids used in this study can be found in Tables 3 and 4. *P. aeruginosa* strains were grown on Luria-Bertaini (LB) medium at 37 °C supplemented as appropriate with 30 µg ml^−1^ gentamicin, 25 µg ml^−1^ irgasan, 5% (w/v) sucrose, 1.0 mM IPTG and arabinose at varying concentrations. *E. coli* was grown routinely in LB medium supplemented as appropriate with 15 µg ml^−1^ gentamicin, 50 µg ml^−1^ trimethoprim and 1% rhamnose. Minimal media for *E. coli* growth under nitrogen limitation consisted of 130 mM K_2_HPO_4_, 33.8 mM KH_2_PO_4_, 5.74 mM K_2_SO_4_, 0.41 mM MgSO_4_ and 0.4% d-glucose, and was supplemented with 2 mM (low) or 10 mM (high) NH_4_Cl as the sole nitrogen source^34,37^. *S. aureus* was grown in tryptic soy broth (TSB) supplemented as appropriate with 10 µg ml^−1^ chloramphenicol and 50 µg ml^−1^ kanamycin.

### Plasmid construction

Details of plasmid construction and primer sequences are provided in Tables 6 and 7. Plasmid pEXG2 was used to make the in-frame deletion constructs pEXG2-∆*gcsR* and pEXG2-Δ*fleQ*, as well as the VSV-G insertion constructs pEXG2-*gcsR–V*, pEXG2-*gacA–V* and pEXG2-*fleQ–V*, the *gcvH2* allelic replacement construct pEXG2-*gcvH2*::*gfp-mut3*, and the DddA fusion constructs pEXG2-*gcsR::dddA*, pEXG2-*gacA–dddA* and pEXG2-*fleQ–dddA*^45^. Plasmid pEXG2-∆*gcsR* and pEXG2-Δ*fleQ* were constructed by amplification of ~400 bp regions of genomic DNA flanking *gcsR* and *fleQ*, respectively, with primers containing restriction sites, followed by digestion and ligation into pEXG2 that had been digested with the appropriate restriction enzymes. To generate plasmid pEXG2-*gcvH2::gfp-mut3*, primers with 3′ overlapping regions were used to amplify *gfp-mut3* from RP1868^46,47^ as well as ~400 bp regions flanking *gcvH2*. Gibson assembly^48^ was used to generate the final construct. C-terminal VSV-G insertion constructs for GcsR–V, GacA–V and FleQ–V were made by amplifying ~400 bp regions flanking each insertion site using primers that contained an in-frame sequence encoding the VSV-G epitope tag. Constructs for generating DddA fusions encoded a protein in which DddA was fused to the C terminus via a 32aa linker (SGGSSGGSSGSETPGTSESATPESSGGSSGGS). To generate these constructs, primers with 3′ overlapping regions were used to amplify both the linker and *dddA*, as well as 500 bp regions flanking the C terminus of each gene. Gibson assembly^48^ was then used for the generation of the pEXG2 plasmids containing each construct, and assembly mixes were transformed into *E. coli* DH5α expressing DddA_I_ from pSCrhaB2-*dddA*_*I*_ to avoid DddA-mediated toxicity. Construction of pEXG2-derived plasmids for deletion of *gacS, retS* and *ung* was previously described^15,49,50^. Site-specific chromosomal insertions of the immunity gene *dddA*_*I*_ (with or without a FLAG tag encoded at the C terminus) were generated using pUC18T-miniTn7T-Gm-pBAD-*araE*^51^. The genes encoding DddA_I_ or DddA_I_-FLAG were amplified and cloned into the KpnI/HindIII sites of this vector through Gibson assembly to generate pUC18-miniTn7T-Gm-pBAD-araE-*dddA*_*I*_ and pUC18T-miniTn7T-Gm-pBAD-araE-*dddA*_*I*_–*F*. The *E. coli* fusion construct pRE112-*ntrC-dddA* was generated by Gibson assembly from plasmid pRE112^52^, and the assembly mix was transformed into *E. coli* EC100D *pir*+ expressing DddA_I_ from pMMB67EH-*dddA*_*I*_.

Plasmids pMMB67EH-*dddA*_*I*_, pMMB67EH-*dddA*_*I*_-FLAG, pPSV39-*dddA*_*I*_, pPSV39-*dddA*_*I*_-FLAG, pBS10-riboE-*dddA*_*I*_ and pBS10-riboE-*dddA*_*I*_-FLAG were made by Gibson assembly, with transformation of assembly mixes into *E. coli* DH5α. To make plasmids pBS10-riboE-dddI and pBS10-riboE-dddI-FLAG, the shuttle vector pAM401-oriT^53^ was first modified by adding the strong constitutive promoter pSpac-hy^54^ and terminator sequences, and by replacement of the Gram-positive chloramphenicol-resistance marker with a kanamycin-resistance marker (*aphA-3*) from pBAVB (Addgene, 65928), generating pBS10. Riboswitch E^55^ and *dddA*_*I*_ (or *dddA*_*I*_-FLAG) were then added by Gibson assembly. Plasmid pEPSA5-dddA was made by Gibson assembly, with transformation of assembly mixes into *E. coli* DH5α expressing immunity from pPSV39-*dddA*_*I*_ before transferring into *S. aureus*. These plasmids will be deposited in Addgene to facilitate maximum availability to the research community. While the availability of these tools should lower the up-front investment for initiating a 3D-seq study, we aim to make additional reagents available to the community in the future. For example, we envision incorporating Ugi and DBP–DddA fusion expression into a single plasmid under the control of orthogonal inducers. This would eliminate the need for chromosomal manipulations, thus making the 3D-seq pipeline faster and more tractable in bacteria without robust genetics.

### Strain construction

*P. aeruginosa* strains containing in-frame deletions of *gcsR, ung, retS or gacS* were constructed by allelic replacement using the appropriate pEXG2-derived deletion construct, and verified by PCR and site-specific or genomic sequencing as described previously^45^. *P. aeruginosa* cells synthesizing GcsR with a C-terminal VSV-G epitope tag from the native chromosomal location were made by allelic replacement using vector pEXG2-GcsR–V. *P. aeruginosa* ∆*retS* mutant cells synthesizing GacA with a C-terminal VSV-G epitope tag from the native chromosomal location (*P. aeruginosa* ∆*retS* GacA–V) were made by allelic replacement using vector pEXG2-GacA–V. The *P. aeruginosa* ∆*gcsR*, GcsR–V and ∆*retS* GacA–V strains were verified by PCR and production of the GcsR–V and GacA–V fusion proteins was verified by western blotting using an antibody against the VSV-G epitope tag. *P. aeruginosa* strains bearing *gfp-mut3* in place of *gcvH2* were generated by two-step allelic replacement using pEXG2-*gcvH2::gfp-mut3* and verified by PCR. *P. aeruginosa* strains producing DddA fusion proteins were generated by first engineering the parent strain to express DddA_I_ or DddA_I_–F from the chromosome under arabinose-inducible control by introduction of pUC18T-miniTn7T-Gm-pBAD-araE-dddA_*I*_ or pUC18T-miniTn7T-Gm-pBAD-araE-*dddA*_*I*_–*F* and helper plasmids pTNS3 and pRK2013 via tetraparental mating^51^. After chromosomal integration, the GmR marker was removed from these cassettes by Flp/FRT recombination using plasmid pFLP2, which was then cured by sucrose counterselection^56^. *P. aeruginosa* strains synthesizing GcsR–DddA, GacA–DddA or FleQ–DddA from the native chromosomal loci of each regulator were then generated by two-step allelic exchange using the relevant pEXG2 construct. Rhamnose (0.1% for *E. coli*) or arabinose (0.1% for *P. aeruginosa*) were maintained during the DddA-fusion-expressing strain construction process to minimize DddA toxicity and off-target activity. Fusion-expressing strains were verified by PCR and by assembly of complete genome sequences obtained during 3D-seq analyses.

The *ung* gene was deleted from *E. coli* MG1655 by one-step allelic exchange^57^ using lambda Red helper plasmid pKD46 and a PCR product amplified from plasmid pKD4 with primers ung_del-F and ung_del-R. The KanR marker within the replacement allele was removed by Flp/FRT recombination using plasmid pFLP2, which was then cured by sucrose counterselection. To generate a strain of *E. coli* able to synthesize NtrC–DddA from the native chromosomal locus, MG1655 Δ*ung* was first transformed with pMMB67EH-*dddA*_*I*_–F for inducible immunity protein expression, and the *ntrC* gene was then replaced by two-step allelic exchange^52^ using plasmid pRE112-*ntrC*–*dddA* introduced by conjugation from S17-1 λpir (which also contained pPSV39-*dddA*_*I*_ to prevent toxicity in the donor strain). IPTG (1 mM) was maintained throughout the construction process to induce DddA_I_ expression, thus minimizing DddA toxicity and off-target activity. Strains were verified by PCR and by assembly of complete genome sequences obtained during 3D-seq analysis.

### Assessing the functionality of the GcsR–DddA fusion protein

To determine the functionality of the GcsR–DddA fusion protein, cells were grown in biological triplicate in No Carbon E (NCE) minimal media^58^ containing arabinose (1%) and glycine (20 mM), or arabinose (1%) and succinate (20 mM), at 37 °C with aeration for 48 h. Growth was determined by measuring the culture OD_600_.

### 3D-seq sample preparation and sequencing

#### Culturing of DddA-fusion-expressing strains

To generate genomic DNA for 3D-seq analysis, *P. aeruginosa* strains carrying specific DddA fusion constructs and attTn7::araC-P_BAD_-*dddA*_*I*_ (GcsR) or attTn7::araC-P_BAD_-*dddA*_*I*_*-F* (GacA, FleQ) were grown for varying amounts of time and with variable levels of arabinose to induce DddA_I_ or DddA_I_–F expression and/or IPTG to induce UGI production from pPSV39-UGI. In each case, the strains were initially streaked for single colonies on LB containing 0.1% or 1% arabinose, and single colonies were used to inoculate quadruplicate liquid cultures containing 0.1% or 1% arabinose. After ~16 h of growth, these cultures were then washed with LB and used to inoculate fresh cultures. For GcsR–DddA in Δ*ung* and *ung*+ backgrounds and for the Δ*ung* strain without a dddA-fusion construct, washed cultures were inoculated into LB containing 0.1% (negative control) or no (experimental) arabinose at OD_600_ = 0.02, then grown for 8 h before diluting back to OD_600_ = 0.02. After an additional ~16 h, cultures were again washed and diluted to OD_600_ = 0.02, then grown a final 8 h before samples were collected for genomic DNA preparation. For *gacA–dddA* (with Δ*retS* or Δ*gacS*) and *fleQ–dddA*, washed cultures were inoculated into LB containing 0.0005% arabinose at OD_600_ = 0.02, then grown for 6.5 h before samples were collected for genomic DNA preparation.

For 3D-seq analysis of NtrC in *E. coli* grown under nitrogen limitation, strain MG1655 Δ*ung* pMMB67EH-*dddI*–F *ntrC*–*dddA* was initially grown for ~16 h as quadruplicate liquid cultures in minimal media supplemented with 10 mM NH_4_Cl, 5 mM l-glutamate and 1 mM IPTG. The cultures were washed with base minimal media (no nitrogen), then diluted to OD_600_ = 0.02 in minimal media lacking IPTG and containing 2 mM NH_4_Cl as the sole nitrogen source. The cultures were grown for ~12 h, reaching saturation, then similarly passaged five additional times by back-diluting to OD_600_ = 0.02 in the same media and growing for ~12 h. After the sixth passage, samples were collected for genomic DNA preparation.

#### Genomic DNA preparation and sequencing

Genomic DNA was isolated from bacterial pellets using DNEasy blood and tissue kit (Qiagen). Sequencing libraries for whole-genome sequencing were prepared from 200–300 ng of DNA using DNA prep kit (Illumina), with KAPA HiFi Uracil+ kit (Roche) used in place of Enhanced PCR Mix for the amplification step. Libraries were sequenced in multiplex by paired-end 150 bp reads on NextSeq 550 and iSeq instruments (Illumina).

### ChIP-seq sample preparation and library construction

A recently published ChIP-seq study employing over-expressed GacA and FleQ identified an exceptionally large number of binding sites for each regulator (1,125 for GacA and 160 for FleQ), yet failed to detect one or more known binding sites for each protein^28^. Given the design of this study and its discrepancies with the extensive published literature for these regulators and with our own 3D-seq results, we performed ChIP-seq analysis in-house with VSV-G-tagged versions of each regulator expressed from their native chromosomal loci. Cultures (200 ml) of the *P. aeruginosa* GcsR–V, WT, ∆*retS*, ∆*retS* GacA–V and FleQ–V strains were grown in biological triplicate to an OD_600_ of 1.5 in LB at 37 °C with aeration. A volume of 80 ml of culture was crosslinked with formaldehyde (1%) for 30 min at room temperature with gentle agitation. Crosslinking was quenched by the addition of glycine (250 mM) and cells were incubated at room temperature for 15 min with gentle agitation. Cells were pelleted by centrifugation, washed three times with phosphate buffered saline and stored at −80 °C before subsequent processing. Cell pellets were resuspended in 1 ml buffer 1 (20 mM KHEPES, pH 7.9, 50 mM KCl, 0.5 mM dithiothreitol, 10% glycerol) plus protease inhibitor (complete-mini EDTA-free (Roche); 1 tablet per 10 ml), diluted to a total volume of 5.2 ml and divided equally among four 15 ml conical tubes (Corning). Cells were subsequently lysed and DNA sheared in a Bioruptor water bath sonicator (Diagenode) by exposure to two 8 min cycles (30 s on, 30 s off) on high setting. Cellular debris was removed by centrifugation at 4 °C for 20 min at 20,000 × *g*. Cleared lysates were adjusted to match the composition of the immunoprecipitation (IP) buffer (10 mM Tris-HCl, pH 8.0, 150 mM NaCl, 0.1% NP-40 alternative (EMD-Millipore, 492018)). The adjusted lysates were combined with anti-VSV-G agarose beads (Sigma) that had been washed once with IP buffer and reconstituted to a 50/50 bead/buffer slurry. For IP, 75 µl of the washed anti-VSV-G beads were added to each of the four aliquots for a given sample. IP was performed overnight at 4 °C with gentle agitation. Beads were then washed 5 times with 1 ml IP buffer and 2 times with 1X TE buffer (10 mM Tris-HCl, pH 7.4, 1 mM EDTA). Immune complexes were eluted from beads by adding 150 µl of TES buffer (50 mM Tris-HCl, pH 8.0, 10 mM EDTA, 1% sodium dodecyl sulfate (SDS)) and heating samples to 65 °C for 15 min. Beads were pelleted by centrifugation (5 min at 16,000 × *g*) at room temperature and a second elution was performed with 100 µl of 1X TE + 1% SDS. Supernatants from both elution steps were combined and incubated at 65 °C overnight to allow crosslink reversal. DNA was then purified with a PCR purification kit (QIAGEN), eluted in 55 µl of 0.1X elution buffer and quantified on an Agilent Bioanalyzer. ChIP-seq libraries were prepared from 1–40 ng of DNA using the NEBNext Ultra II DNA library prep kit for Illumina (NEB). Adaptors were diluted 10-fold before ligation. AMPure XP beads (Beckman Coulter) were used to purify libraries, which were subjected to 7 rounds of amplification without size selection. Libraries were sequenced by the Biopolymers Facility (Harvard Medical School) on an Illumina NextSeq 500 producing 75 bp paired-end reads^59^.

### ChIP-seq data analysis

ChIP-seq data were analyzed as described previously^59^. Paired-end reads corresponding to fragments of 200 bp or less were mapped to the PAO1 genome (NCBI RefSeq NC_002516) using Bowtie2 version 2.3.4.3^60^. Only read 1 from each pair of reads was extracted and regions of enrichment were identified using QuEST version 2.4^61^. Reads collected from the PAO1 replicates (that is, IP from PAO1 cells that do not synthesize any VSV-G-tagged protein) were merged and served as the mock control for the reads from each of the PAO1 GcsR–V and PAO1 FleQ–V replicates. Merged reads from the PAO1 ∆*retS* replicates served as the mock control for the reads from the PAO1 ∆*retS* GacA–V replicates. The mock control data were used to determine the background for each corresponding ChIP biological replicate. The following criteria were used to identify regions of enrichment (peaks): (1) they must be 5-fold enriched in reads compared with the background, as recommended by Landt et al.^62^; (2) they are not present in the mock control; (3) they have a positive peak shift and strand correlation; and (4) they have a *q*-value of less than 0.01. Peaks of enrichment for GcsR–V and GacA–V were defined as the maximal region identified in at least two biological replicates. Data were visualized using the Integrative Genomics Viewer (IGV) version 2.5.0^63^. Peak analyses used BEDtools version 2.27.1.

### 3D-seq data analysis

Fastq reads were first pre-processed using the HTStream pipeline v. 1.3.0 (https://s4hts.github.io/HTStream/), where the serial pipeline is hts_SuperDeduper → hts_SeqScreener → hts_AdapterTrimmer → hts_QWindowTrim → hts_LengthFilter → hts_Stats. In each case, logging was enabled and default settings were used, with the following exceptions: (1) for hts_QWindowTrim, a window size of 20 bp was used with a minimum quality score of 10; (2) for hts_LengthFilter, the minimum length was set to half the mean read length. Reads were subsequently aligned to the PAO1 UW reference sequence (https://www.ncbi.nlm.nih.gov/nuccore/NC_002516.2) using Minimap2 v. 2.17-r974-dirty (https://lh3.github.io/minimap2/) and the alignments were processed into sorted BAM files with SAMTools v. 1.10 (https://www.htslib.org/). Alignment position read base counts were then enumerated using the PySAM v. 0.16.0.1 (https://pysam.readthedocs.io/en/latest/) count_coverage function, with these settings: read_callback = ´all´, quality_threshold = 20. The reference genome was then surveyed using Biopython v. 1.78 (https://biopython.org/) to determine the proportion of high-quality read pairs covering each 5´-TC-3´ site (the preferred DddA target sequence context^13^; on either strand that showed the alternative sequence 5´-TT-3´ (representing cytidine deamination), and corresponding base counts and allele frequencies were tabulated using Pandas v. 1.3.0. (https://pandas.pydata.org/).

To generate minimally filtered datasets, sites with sequence coverage of less than 15 read pairs for that sample and sites with >95% C•G-to-T•A transition frequency in any individual replicate of a given sample were ignored, as were a set of 52 sites within a phage region known to display hypervariability^64^ in the case of *P. aeruginosa*. Average C•G-to-T•A transition frequency was then calculated using remaining positions for each set of quadruplicate samples per condition. To generate more stringently filtered data, the mean C•G-to-T•A transition frequency was calculated for each position at which 3 of 4 replicate samples for a given condition exhibited at least 1 sequencing read containing the mutation. Data passing these criteria were then used for statistical analyses. To generate the representations of the data shown in Fig. 3, these data were further processed by the calculation of a moving average employing a 75 bp window.

3D-seq results (and ChIP-seq results) were benchmarked for accuracy using the location of known binding motifs for GcsR, GacA and FleQ. To identify the locations of FleQ binding motifs in relation to binding sites detected, the regions encompassing peaks detected by each method were searched with MEME V. 5.4.1 using parameters tailored to the 14 bp FleQ binding motif identified using DNase I footprinting^25^. This led to the identification of one or more copies of a motif within the peak region for 13/14 peaks detected by 3D-seq and 29/40 peaks detected by ChIP-seq, with these copies sharing a consensus sequence and localization pattern with that of the binding motif previously identified^25^. The *P* value for all detected instances of the motif was <1.0 × 10^−5^.

### Statistical analysis

We divided the analysis into two steps: peak detection and peak-parameter inference. In the peak detection step, we used a canonical frequentist approach: null hypothesis testing to determine the number and approximate position of the peaks in the data. Then, in a second step, we optimized the model parameters describing each peak individually using a slower but more accurate numerical Maximum Likelihood Estimation to optimize peak-parameter inference (see Supplementary Note 1 for full details).

### Quantitative reverse-transcriptase PCR

*P. aeruginosa* WT and Δ*fleQ* cultures were back-diluted from overnight cultures to OD_600_ = 0.01 in 5 ml LB and grown at 37 °C with shaking to early stationary phase (OD_600_ ≈ 2). At this time, 4 ml of cells were collected and total RNA was isolated using Tri-Reagent (Millipore Sigma) according to the manufacturer’s recommendations. Complementary DNA synthesis was performed using SuperScript III reverse transcriptase (Invitrogen) and quantitative reverse-transcriptase PCR (qRT-PCR) was performed as described previously^65^ using a LightCycler 96 system (Roche). The abundances of transcripts were measured relative to the abundance of the *clpX* transcript. qRT-PCR was performed twice on sets of biological triplicates. Relative expression values were calculated using the comparative threshold cycle (CT) method (2^-ΔΔCT)^66^. Results were analyzed using a two-tailed Student’s *t*-test.

### DddA_I_ expression plasmid testing

To evaluate titration of DddA_I_ activity when expressed from pPSV39 in *E. coli*, cultures of strain MG1655 bearing pSCrhaB2-*dddA* and pPSV39-*dddA*_*I*_ were grown overnight in LB with gentamycin and 1 mM IPTG, washed and diluted to OD_600_ = 0.02 in media lacking IPTG, then grown to OD_600_ = ~0.6, divided and supplemented with IPTG at various levels. After 20 min of incubation at 37 °C with agitation, the cultures were supplemented with either water or rhamnose (1% final concentration) and further incubated at 37 °C with agitation. Viable titres were assessed by serial dilution and plating on media with 1 mM IPTG at the time of rhamnose addition and subsequently at ~60 and 120 min. To evaluate titration of DddA_I_ activity when expressed from pBS10-riboE in *S. aureus*, cultures of strain JE2 bearing pEPSA5-dddA and pBS10-riboE-*dddA*_*I*_ were grown to late log phase in no-dextrose TSB with chloramphenicol and kanamycin (TSB-CK) supplemented with 1 mM theophylline, washed and resuspended to a starting OD_600_ of 0.25 in TSB-CK supplemented with 0.2% xylose (or water) and theophylline at various concentrations (see Supplementary Fig. 5 legend). The cultures were incubated at 37 °C with agitation and viable titres were assessed at 0, 60 and 120 min by serial dilution and plating on tryptic soy agar with chloramphenicol, kanamycin and 1 mM theophylline.

### Materials availability

Plasmids generated in this study with utility for future 3D-seq applications have been deposited in Addgene.

### Reporting summary

Further information on research design is available in the Nature Research Reporting Summary linked to this article.

## Supplementary information

Supplementary Information Supplementary Tables 1-7 and Note 1.

Reporting Summary

## Data Availability

Sequence data associated with this study are available from the Sequence Read Archive at BioProject PRJNA748760. Publicly available datasets employed in this study include the *Pseudomonas aeruginosa* PAO1 UW reference sequence (NCBI accession NC_002516.2) and the *Escherichia coli* K-12 MG1655 reference sequence (NCBI accession NC_000913.3). Source data are provided with this paper.

## Source data

Source Data Fig. 1 Values used for generating graphs.
Source Data Fig. 2 Values used for generating graphs.
Source Data Fig. 3 Values used for generating graphs.
Source Data Fig. 4 Values used for generating graphs.
Source Data Fig. 5 Values used for generating graphs.
Source Data Fig. 6 Values used for generating graphs.
Source Data Extended Data Fig. 1 Values used for generating graphs.
Source Data Extended Data Fig. 2 Values used for generating graphs.
Source Data Extended Data Fig. 4 Values used for generating graphs.
Source Data Extended Data Fig. 5 Values used for generating graphs.
Source Data Extended Data Fig. 6 Values used for generating graphs.

## Code availability

Computer code generated for this study is available from GitHub at https://github.com/marade/3DSeqTools, version dd7ca68.
