# Supplementary material for: Genome-wide protein–DNA interaction site mapping in bacteria using a double-stranded DNA-specific cytosine deaminase
Source: Nat Microbiol. 2022 Jun 1;7(6):844–55. doi: 10.1038/s41564-022-01133-9 (PMC9159945; doi:10.1038/s41564-022-01133-9)
Supplement: Supplementary file 1 — Supplementary Tables 1-7 and Note 1. [file 41564_2022_1133_MOESM1_ESM.pdf]

nature portfolio

https://doi.org/10.1038/s41564-022-01133-9

Supplementary information

Genome-wide protein–DNA interaction site mapping in bacteria using a double-stranded DNA-specific cytosine deaminase

In the format provided by the authors and unedited

Supplementary Table 1. Significant peaks detected in this study by 3D-seq.

Peak number[1]	p-value[2]	Peak maximum position	Peak height[3]	Width[4]	Closest annotated gene	Distance to closest ChIP-seq peak[5]
GcsR (Δ*ung* Tn7::pAra-$dddA_I$ *gcsR–dddA*)						
1	<1.9E-99	2747212	0.0308	650	*gcvH2*	229
2	4.4E-6	2754622	0.0032	507	*gcvH2*	ND
GacA (Δ*ung* Δ*retS* Tn7::pAra-$dddA_I$ *gacA–dddA*)						
1	2.0E-27	586705	0.0096	552	*rsmY*	278
2	4.5E-17	4057755	0.0090	449	*rsmZ*	53
GacA (Δ*ung* Δ*gacS* Tn7::pAra-$dddA_I$ *gacA–dddA*)						
None detected.						
FleQ (Δ*ung* Tn7::pAra-$dddA_I$ *fleQ–dddA*)						
1	<1.9E-99	5192977	0.0182	828	*cdrA*	519
2	<1.9E-99	2453477	0.0196	624	*pslA*	45
3	4.0E-25	3221641	0.0092	479	PA2869	10
4	5.9E-25	1549819	0.0079	402	*bdlA*	219
5	1.6E-21	3434343	0.0067	704	*pelA*	289
6	2.2E-16	1592133	0.0066	563	*motD*	28
7	2.2E-16	1187368	0.0042	480	*fleQ*	245
8	5.7E-14	2129043	0.0054	529	PA1945	209
9	2.4E-11	196854	0.0070	439	*siaA*	344
10	9.9E-11	1582818	0.0036	429	*flhF*	204
11	2.2E-09	2737599	0.0075	686	PA2440	109
12	1.4E-04	1572170	0.0030	394	*fliL*	177
13	1.6E-02	3751994	0.0027	316	PA3340	14
14	1.8E-02	1215200	0.0040	508	*yfiR*	27
GcsR (Tn7::pAra-$dddA_I$ *gcsR–dddA* pPSV39::pLac-*ugi*)						
1	<1.9E-99	2747582	0.0830	511	*gcvH2*	141
2	<1.9E-99	6199779	0.0391	610	PA5508	ND
3	2.1E-83	2744696	0.0344	204	*gcvH2*	ND
4	1.5E-26	2141807	0.0153	1000	PA1957	ND
5	5.0E-22	6151145	0.0104	934	PA5459	ND
6	1.0E-21	4230516	0.0117	756	PA3372	ND
7	1.8E-21	2754908	0.0127	283	PA2454	ND
8	2.8E-17	1121658	0.0117	612	PA1032	ND
9	4.0E-15	2749090	0.0463	26	PA2448	ND
10	1.5E-12	6195909	0.0110	364	PA5503	ND
11	9.0E-12	2751477	0.0155	213	PA2449	ND
12	1.5E-09	2742380	0.0107	166	PA2443	ND
13	8.9E-08	2750504	0.0168	109	*gcsR*	ND

Supplementary Table 2. Significant peaks detected in this study by ChIP-seq.

E coli NtrC (Δ*ung ntrC-dddA* pMMB67EH-$dddA_I$-FLAG)						Brown[6]	Aquino[7]
1	<1.9E-99	848143	0.026	438	*glnH*	65	34
2	<1.9E-99	4058196	0.014	300	*glnA*	118	40
3	7.82E-33	4056349	0.006	379	*glnL*	49	14
4	1.70E-28	895046	0.005	479	*potG*	ND	78
5	1.11E-16	2914513	0.003	388	*rlmD (relA)*[8]	154	64
6	2.38E-14	2427052	0.004	425	*argT (hisJ)*	827[9]	29
7	1.58E-06	472363	0.003	480	*mdlB (glnK)*	194	120
8	7.74E-04	689269	0.002	432	*gltL*	71	63
	>0.01[10]	708971	0.001	409	*chiP*	ND	33
	>0.01	700726	0.001	391	*nagC (umpH)*	ND	209
	>0.01	1908217	0.001	409	*yobF*	ND	23
	>0.01	1074189	0.001	408	*rutA*	151	17
	>0.01	2304574	0.001	396	*eco*	ND	ND
	>0.01	1272816	0.001	415	*chaC*	ND	45
	>0.01	4052559	0.001	439	*hemN*	ND	ND
	>0.01	4054538	0.001	127	*glnG*	ND	ND
	>0.01	690327	<0.001	360	*lnt*	ND	ND
	>0.01	845464	<0.001	384	*ybiO*	ND	ND
	>0.01	1866051	0.001	397	*mipA*	ND	ND
	>0.01	2428027	<0.001	381	*argT*	56	56

[1]Peaks are listed in in order of increasing p-value.

[2]P-values calculated using the likelihood ratio test developed in this study, described in Supplementary Note 1.

[3,4] The peak profile function represents cell-mean allele frequency as a function of genomic position. The amplitude parameter I represents the height of the peak of the profile function. The width parameter L controls its width. See Supplementary Note 1.

[5]Distance to the nearest significant peak detected by ChIP-seq in our study or the indicated published reports was calculated using the peak maximum location for each method. Not detected (ND) is listed when no peak was present within 1000 bp.

[6,7]Distance to nearest ChIP-seq peak reported by the indicated studies (Brown *et. al.*[1]*;* Aquino *et al.*[2]).

[8]When peaks fell within a genic region upstream of a previously confirmed NtrC target, the latter is provided in parantheses.

[9]This peak is localized in the intergenic region upstream of *argT*, while the peaks detected by 3D-seq and the Aquino *et al.* study are localized in the 3' end of *argT* and upstream of *hisJ*.

[10]Non-significant peaks detected by the 3D-seq peak calling algorithm are listed for the purposes of comparison to 3D-seq (see text).

Peak number[1]	Fold enrichment	Peak maximum	Peak start	Peak end	Closest annotated gene
GcsR (*gcsR*–VSV-G)					
1	92	2747441	2746047	2748292	*gcvH2*
2	13	5402788	5402462	5403174	*lipC*
3	9.1	945626	945487	945780	PA0864
4	5.6	2516215	2516073	2516373	PA2288
GacA (Δ*retS gacA*–VSV-G)					
1	215	586983	586145	587484	*rsmY*
2	212	4057808	4057399	4058161	*rsmZ*
FleQ (*fleQ*–VSV-G)					
1	249	2453522	2452032	2454624	*pslA*
2	195	5193496	5191924	5194398	*hprA*
3	121	3434054	3433311	3434803	*pelA*
4	109	197198	195720	198717	PA0172
5	63.1	2129252	2128523	2129734	PA1945
6	56.7	4831221	4830146	4832283	*pctC*
7	49.0	2737708	2737011	2738270	PA2440
8	45.8	5820572	5820016	5821653	*arcD*
9	42.0	1550038	1549058	1551009	PA1424
10	41.6	1187613	1186221	1188580	*fleQ*
11	34.4	3751980	3751110	3752837	PA3340
12	27.4	3221651	3221084	3222288	PA2869
13	25.9	2858395	2857908	2858862	PA2531
14	25.4	204620	203838	205074	PA0179
15	22.0	2926537	2925610	2926963	*gacA*
16	21.0	5067832	5066935	5068210	PA4523
17	18.1	1592161	1591586	1592430	PA1462
18	17.9	3791236	3790895	3792351	*amrZ*
19	17.1	1582614	1581703	1583031	*flhF*
20	16.3	3754992	3754364	3755298	PA3343
21	15.6	1571993	1571773	1572616	PA1442
22	11.7	3567509	3567229	3568007	PA3177
23	11.5	1185011	1184404	1185494	*fliD*
24	10.8	3762086	3761425	3763135	PA3350
25	10.7	1406779	1406573	1407206	PA1296
26	10.4	2390201	2389881	2390419	PA2166
27	10.2	1580264	1580028	1580446	*flhA*
28	7.81	4215154	4214936	4215372	PA3762

29	7.45	1192018	1191843	1192218	*fliE*
30	7.30	4748681	4748496	4748873	*uvrA*
31	5.93	2010938	2010801	2011082	PA1851
32	5.82	3315728	3315596	3315862	PA2955
33	5.66	1039144	1038536	1039804	PA0951a
34	5.63	205966	205581	206201	*cttP*
35	5.59	1191610	1191437	1191747	*fliE*
36	5.53	3195799	3195616	3196007	PA2841
37	5.35	1168241	1168099	1168376	*flgG*
38	5.30	1060664	1060099	1060829	PA0976.1
39	5.22	1215173	1215006	1215348	*yfiR*
40	5.20	4152467	4152158	4152647	*wspA*

[1]Peaks are listed in order of decreasing fold enrichment. The following criteria were used to identify significant regions of enrichment (peaks): (i) they must be 5-fold enriched in reads compared to the background, as recommended by Landt *et al.*[3], (ii) they are are not present in the mock control, (iii) they have a positive peak shift and strand correlation, and (iv) they have a q-value of less than 0.01.

Supplementary Table 3. Location and number of SNPs detected in individual clones following expression of GcsR-DddA.

Clone ID	Method[1]	n SNPs	SNP locations[2]
W001	WGS	0	
W002	WGS	0	
W003	WGS	1	2747005
W004	WGS	0	
W005	WGS	0	
W006	WGS	0	
W007	WGS	0	
W008	WGS	0	
W009	WGS	0	
W010	WGS	0	
W011	WGS	0	
W012	WGS	0	
W013	WGS	3	2747027, 2747501, 2747636
W014	WGS	0	
W015	WGS	0	
W016	WGS	0	
W017	WGS	0	
W018	WGS	0	
W019	WGS	0	
W020	WGS	0	
W021	WGS	0	
W022	WGS	0	
W023	WGS	0	
W024	WGS	0	
W025	WGS	1	2747245
W026	WGS	0	
W027	WGS	0	
W028	WGS	0	
W029	WGS	0	
W030	WGS	0	
W031	WGS	0	
W032	WGS	0	
W033	WGS	0	
W034	WGS	0	
W035	WGS	0	
W036	WGS	0	
W037	WGS	0	
W038	WGS	0	
W039	WGS	0	
W040	WGS	0	
W041	WGS	0	
W042	WGS	0	

W043	WGS	0	
W044	WGS	0	
W045	WGS	0	
W046	WGS	0	
W047	WGS	0	
W048	WGS	0	
W049	WGS	0	
W050	WGS	0	
W051	WGS	1	2747381
W052	WGS	0	
W053	WGS	0	
W054	WGS	0	
W055	WGS	0	
W056	WGS	0	
W057	WGS	0	
W058	WGS	0	
W059	WGS	0	
W060	WGS	0	
W061	WGS	0	
W062	WGS	0	
W063	WGS	0	
W064	WGS	0	
W065	WGS	0	
W066	WGS	0	
W067	WGS	0	
W068	WGS	0	
W069	WGS	0	
W070	WGS	0	
W071	WGS	0	
W072	WGS	0	
W073	WGS	0	
W074	WGS	0	
W075	WGS	1	2747205
W076	WGS	6	2746942, 2747109, 2747114, 2747245, 2747275, 2747326
W077	WGS	0	
W078	WGS	0	
W079	WGS	0	
W080	WGS	0	
W081	WGS	0	
W082	WGS	0	
W083	WGS	0	
W084	WGS	0	
Sa001	Sanger	0	
Sa002	Sanger	0	
Sa003	Sanger	0	

Sa004	Sanger	9	2747244, 2747245, 2747275, 2747383, 2747431, 2747441, 2747464, 2747518, 2747575
Sa005	Sanger	0	
Sa006	Sanger	0	
Sa007	Sanger	1	2747518
Sa008	Sanger	0	
Sa009	Sanger	0	
Sa010	Sanger	1	2747005
Sa011	Sanger	0	
Sa012	Sanger	1	2747326
Sa013	Sanger	1	2747028
Sa014	Sanger	0	
Sa015	Sanger	1	2747569
Sa016	Sanger	3	2747005, 2747436, 2747437
Sa017	Sanger	0	
Sa018	Sanger	0	
Sa019	Sanger	0	
Sa020	Sanger	0	
Sa021	Sanger	0	
Sa022	Sanger	0	
Sa023	Sanger	0	
Sa024	Sanger	0	
Sa025	Sanger	0	
Sa026	Sanger	2	2747244, 2747245
Sa027	Sanger	0	
Sa028	Sanger	0	
Sa029	Sanger	2	2747291, 2747292
Sa030	Sanger	0	
Sa031	Sanger	0	
Sa032	Sanger	0	
Sa033	Sanger	2	2747025, 2747043
Sa034	Sanger	0	
Sa035	Sanger	1	2747275
Sa036	Sanger	0	
Sa037	Sanger	0	
Sa038	Sanger	0	
Sa039	Sanger	0	
Sa040	Sanger	0	
Sa041	Sanger	2	2747090, 2747106
Sa042	Sanger	0	
Sa043	Sanger	0	
Sa044	Sanger	0	
Sa045	Sanger	0	
Sa046	Sanger	0	
Sa047	Sanger	0	
Sa048	Sanger	0	

Sa049	Sanger	3	2747204, 2747205, 2747383
Sa050	Sanger	0	
Sa051	Sanger	0	
Sa052	Sanger	0	
Sa053	Sanger	0	
Sa054	Sanger	1	2747028
Sa055	Sanger	0	
Sa056	Sanger	0	
Sa057	Sanger	0	
Sa058	Sanger	0	
Sa059	Sanger	2	2747204, 2747205
Sa060	Sanger	0	
Sa061	Sanger	0	
Sa062	Sanger	0	
Sa063	Sanger	0	
Sa064	Sanger	3	2747204, 2747205, 2747431
Sa065	Sanger	2	2747569, 2747575
Sa066	Sanger	0	
Sa067	Sanger	0	
Sa068	Sanger	0	
Sa069	Sanger	1	2747395
Sa070	Sanger	0	
Sa071	Sanger	0	
Sa072	Sanger	0	
Sb001	Sanger	0	
Sb002	Sanger	0	
Sb003	Sanger	2	2747204, 2747205
Sb004	Sanger	0	
Sb005	Sanger	0	
Sb006	Sanger	0	
Sb007	Sanger	0	
Sb008	Sanger	0	
Sb009	Sanger	0	
Sb010	Sanger	0	
Sb011	Sanger	0	
Sb012	Sanger	4	2747005, 2747109, 2747204, 2747205
Sb013	Sanger	0	
Sb014	Sanger	0	
Sb015	Sanger	0	
Sb016	Sanger	0	
Sb017	Sanger	0	
Sb018	Sanger	0	
Sb019	Sanger	0	
Sb020	Sanger	0	
Sb021	Sanger	1	2747383
Sb022	Sanger	0	

Sb023	Sanger	0	
Sb024	Sanger	0	
Sb025	Sanger	0	
Sb026	Sanger	1	2747305
Sb027	Sanger	0	
Sb028	Sanger	0	
Sb029	Sanger	2	2747244, 2747245
Sb030	Sanger	0	
Sb031	Sanger	2	2747028, 2747382
Sb032	Sanger	0	
Sb033	Sanger	0	
Sb034	Sanger	0	
Sb035	Sanger	0	
Sb036	Sanger	0	
Sb037	Sanger	0	
Sb038	Sanger	3	2747437, 2747643, 2747644
Sb039	Sanger	0	
Sb040	Sanger	0	
Sb041	Sanger	0	
Sb042	Sanger	0	
Sb043	Sanger	1	2747205
Sb044	Sanger	0	
Sb045	Sanger	0	
Sb046	Sanger	1	2747647
Sb047	Sanger	2	2747204, 2747205
Sb048	Sanger	0	
Sb049	Sanger	0	
Sb050	Sanger	0	
Sb051	Sanger	0	
Sb052	Sanger	0	
Sb053	Sanger	0	
Sb054	Sanger	0	
Sb055	Sanger	1	2747319
Sb056	Sanger	1	2747382
Sb057	Sanger	0	
Sb058	Sanger	0	
Sb059	Sanger	0	
Sb060	Sanger	0	
Sb061	Sanger	1	2747084
Sb062	Sanger	0	
Sb063	Sanger	0	
Sb064	Sanger	0	
Sb065	Sanger	0	
Sb066	Sanger	0	
Sb067	Sanger	0	
Sb068	Sanger	0	

Sb069	Sanger	0	
Sb070	Sanger	0	
Sb071	Sanger	0	
Sb072	Sanger	0	
Sc001	Sanger	0	
Sc002	Sanger	0	
Sc003	Sanger	0	
Sc004	Sanger	0	
Sc005	Sanger	2	2747244, 2747245
Sc006	Sanger	0	
Sc007	Sanger	0	
Sc008	Sanger	0	
Sc009	Sanger	0	
Sc010	Sanger	0	
Sc011	Sanger	0	
Sc012	Sanger	1	2747084
Sc013	Sanger	0	
Sc014	Sanger	0	
Sc015	Sanger	0	
Sc016	Sanger	0	
Sc017	Sanger	0	
Sc018	Sanger	5	2747244, 2747245, 2747301, 2747314, 2747315
Sc019	Sanger	0	
Sc020	Sanger	0	
Sc021	Sanger	0	
Sc022	Sanger	0	
Sc023	Sanger	0	
Sc024	Sanger	1	2747475
Sc025	Sanger	0	
Sc026	Sanger	1	2747028
Sc027	Sanger	4	2746971, 2746983, 2747025, 2747463
Sc028	Sanger	0	
Sc029	Sanger	0	
Sc030	Sanger	3	2747244, 2747245, 2747464
Sc031	Sanger	0	
Sc032	Sanger	0	
Sc033	Sanger	1	2747028
Sc034	Sanger	17	2746971, 2746983, 2747028, 2747090, 2747109, 2747123, 2747245, 2747382, 2747401, 2747463, 2747518, 2747580, 2747589, 2747600, 2747616, 2747637, 2747647
Sc035	Sanger	0	
Sc036	Sanger	0	
Sc037	Sanger	0	
Sc038	Sanger	0	
Sc039	Sanger	0	

Sc040	Sanger	0	
Sc041	Sanger	4	2747244, 2747245, 2747431, 2747575
Sc042	Sanger	0	
Sc043	Sanger	0	
Sc044	Sanger	2	2747114, 2747275
Sc045	Sanger	0	
Sc046	Sanger	1	2747617
Sc047	Sanger	0	
Sc048	Sanger	0	
Sc049	Sanger	0	
Sc050	Sanger	0	
Sc051	Sanger	0	
Sc052	Sanger	0	
Sc053	Sanger	0	
Sc054	Sanger	0	
Sc055	Sanger	0	
Sc056	Sanger	0	
Sc057	Sanger	2	2747204, 2747205
Sc058	Sanger	0	
Sc059	Sanger	0	
Sc060	Sanger	0	
Sc061	Sanger	0	
Sc062	Sanger	0	
Sc063	Sanger	1	2747109
Sc064	Sanger	2	2747244, 2747245
Sc065	Sanger	0	
Sc066	Sanger	0	
Sc067	Sanger	6	2746938, 2746983, 2747022, 2747028, 2747357, 2747616
Sc068	Sanger	0	
Sc069	Sanger	0	
Sc070	Sanger	0	
Sc071	Sanger	3	2747204, 2747205, 2747518
Sc072	Sanger	0	
Sd001	Sanger	0	
Sd002	Sanger	0	
Sd003	Sanger	0	
Sd004	Sanger	0	
Sd005	Sanger	0	
Sd006	Sanger	1	2746983
Sd007	Sanger	0	
Sd009	Sanger	0	
Sd010	Sanger	0	
Sd011	Sanger	0	
Sd012	Sanger	0	
Sd013	Sanger	0	

Sd014	Sanger	0	
Sd015	Sanger	0	
Sd016	Sanger	0	
Sd017	Sanger	1	2747637
Sd018	Sanger	0	
Sd019	Sanger	2	2747383, 2747424
Sd021	Sanger	4	2747028, 2747205, 2747291, 2747292
Sd022	Sanger	0	
Sd023	Sanger	0	
Sd025	Sanger	0	
Sd026	Sanger	0	
Sd027	Sanger	0	
Sd028	Sanger	1	2747340
Sd029	Sanger	4	2747109, 2747114, 2747244, 2747245
Sd030	Sanger	0	
Sd031	Sanger	0	
Sd032	Sanger	0	
Sd033	Sanger	0	
Sd034	Sanger	0	
Sd035	Sanger	0	
Sd036	Sanger	0	
Sd037	Sanger	0	
Sd038	Sanger	3	2747109, 2747204, 2747205
Sd039	Sanger	0	
Sd040	Sanger	0	
Sd041	Sanger	0	
Sd042	Sanger	0	
Sd043	Sanger	0	
Sd044	Sanger	0	
Sd045	Sanger	1	2747090
Sd046	Sanger	5	2747078, 2747244, 2747245, 2747431, 2747464
Sd047	Sanger	1	2747245
Sd048	Sanger	0	
Sd049	Sanger	1	2747255
Sd050	Sanger	0	
Sd051	Sanger	1	2747475
Sd052	Sanger	0	
Sd053	Sanger	0	
Sd054	Sanger	2	2747204, 2747205
Sd055	Sanger	0	
Sd056	Sanger	0	
Sd057	Sanger	3	2747005, 2747204, 2747205
Sd058	Sanger	0	
Sd059	Sanger	0	
Sd060	Sanger	0	
Sd061	Sanger	0	

Sd062	Sanger	2	2747244, 2747245
Sd063	Sanger	2	2747245, 2747275
Sd064	Sanger	0	
Sd065	Sanger	0	
Sd066	Sanger	1	2747205
Sd067	Sanger	0	
Sd068	Sanger	0	
Sd069	Sanger	0	
Sd070	Sanger	0	
Sd071	Sanger	0	

[1]WGS, whole genome sequencing. Sanger, targeted amplification and Sanger sequencing of nucleotide positions 2,746,930 to 2,747,667.
[2]For WGS, the location identifies the first top-strand base of the 5'-TC-3' or 5'-GA-3' dinucleotide modified to 5'-TT-3' or 5'-AA-3', respectively. For Sanger sequencing, the location identifies the specific C or G base modified to T or A, respectively. The locations listed for the Sanger method include both single and multiple Cs immediately 3' of a T that were all modified to Ts, and both single and multiple Gs immediately 5' of an A that were all modified to As.

Supplementary Table 4. Bacterial strains used in this study.

Strain	Source
P. aeruginosa **strains**	
PAO1	[4]
Δ*ung*	[5]
attTn7::*araC*-P_{BAD}-*dddA*$_I$	This study
Δ*ung* attTn7::*araC*-P_{BAD}-*dddA*$_I$	This study
attTn7::*araC*-P_{BAD}-*dddA*$_I$ *gcsR-dddA*	This study
Δ*ung* attTn7::*araC*-P_{BAD}-*dddA*$_I$ *gcsR-dddA*	This study
attTn7::*araC*-P_{BAD}-*dddA*$_I$ *gcsR-dddA* pPSV39(empty)	This study
attTn7::*araC*-P_{BAD}-*dddA*$_I$ *gcsR-dddA* pPSV39-*ugi*	This study
attTn7::*araC*-P_{BAD}-*dddA*$_I$-FLAG	This study
Δ*ung* attTn7::*araC*-P_{BAD}-*dddA*$_I$-FLAG	This study
Δ*ung* attTn7::*araC*-P_{BAD}-*dddA*$_I$-FLAG *gacA-dddA*	This study
Δ*ung* attTn7::*araC*-P_{BAD}-*dddA*$_I$-FLAG *gacA-dddA* Δ*retS*	This study
Δ*ung* attTn7::*araC*-P_{BAD}-*dddA*$_I$-FLAG *gacA-dddA* Δ*gacS*	This study
Δ*ung* attTn7::*araC*-P_{BAD}-*dddA*$_I$-FLAG *fleQ-dddA*	This study
Δ*gcsR*	This study
Δ*fleQ*	This study
GcsR-V	This study
Δ*retS*	This study
Δ*retS* GacA-V	This study
gcvH2::gfp-mut3	This study
Δ*gcsR* *gcvH2::gfp-mut3*	This study
E. coli **strains**	
DH5α	
MG1655	
SM10	Novagen
HB101	[6]
EC100D *pir*+	NEB
S17-1 λ*pir*	[7]
IM08B	[8]
MG1655 Δ*ung*	This study
MG1655 Δ*ung* pMMB67EH-*dddA*$_I$-FLAG	This study
MG1655 Δ*ung* pMMB67EH-*dddA*$_I$-FLAG *ntrC-dddA*	This study
MG1655 pSCrhaB2-*dddA* pPSV39-*dddA*$_I$	This study
MG1655 pSCrhaB2-*dddA* pPSV39-*dddA*$_I$-FLAG	This study
S. aureus **strains**	
JE2	[9]
JE2 pEPSA5-*dddA* pBS10-riboE-*dddA*$_I$	This study
JE2 pEPSA5-*dddA* pBS10-riboE-*dddA*$_I$-FLAG	This study

Supplementary Table 5. Plasmids used in this study.

Plasmid	Reference
pEXG2	[10]
pSCrhaB2	[11]
pPSV39	[12]
pUC18T-miniTn7T-Gm-pBAD-araE	[13]
pRK2103	[14]
pTNS3	[15]
pFLP2	[16]
pMMB67EH	[17]
pRMC2	[18]
pORF5-Tnp	[19]
pRE112	[20]
pKD4	[21]
pKD46	[21]
pEXG2-Δ*ung*	[5]
pEXG2-Δ*retS* (pEXG2_ΔPA4856)	[22]
pEXG2-Δ*gacS* (pEXG2_ΔPA0928)	[23]
pPSV39-dddA_I	[24]
pSCrhaB2-*dddA*	[24]
pAM401	[25]
pEPSA5	[26]
pSCrhaB2-dddA_I	This study
pEXG2-*gcsR*-*dddA*	This study
pEXG2-*gacA*-*dddA*	This study
pEXG2-*fleQ*-*dddA*	This study
pEXG2-Δ*gcsR*	This study
pEXG2-GscR-V	This study
pEXG2-GacA-V	This study
pEXG2-FleQ-V	This study
pPSV39-*ugi*	This study
pUC18T-miniTn7T-Gm-pBAD-araE-dddA_I	This study
pUC18T-miniTn7T-Gm-pBAD-araE-dddA_I-FLAG	This study
pRE112-*ntrC*-*dddA*	This study
pMMB67EH-dddA_I	This study
pMMB67EH-dddA_I-FLAG	This study
pPSV39-dddA_I-FLAG	This study
pBS10	This study
pBS10-riboE-dddA_I	This study
pBS10-riboE-dddA_I-FLAG	This study
pEPSA5-dddA	This study

Supplementary Table 6. Plasmid construction.
The following plasmids were generated by combining the individual fragments listed using either Gibson cloning, overlap extension PCR or SLiCE cloning. Primer sequences are listed in Table S7.

pSCrhaB2-*ddd*A_I

Fragment	Template	F primer	R primer
pSCrhaB2/NdeI/XbaI	NA		
dddA_I	*ddd*A_I [24]	pSCRhaB::*ddd*A_I-F	pSCRhaB::*ddd*A_I-R

pEXG2-*gcsR-dddA*

Fragment	Template	F primer	R primer
pEXG2/HindIII/XhoI	NA		
3' end of *gcsR*	PAO1 gDNA	*gcsR-dddA* 1	*gcsR-dddA* 2
linker+*dddA*	XTEN linker [27]	*gcsR-dddA* 3	*gcsR-dddA* 4
gcsR 3' flank	PAO1 gDNA	*gcsR-dddA* 5	*gcsR-dddA* 6

pEXG2-*gacA-dddA*

Fragment	Template	F primer	R primer
pEXG2/HindIII/XhoI	NA		
3' end of *gacA*	PAO1 gDNA	*gacA-dddA* 1	*gacA-dddA* 2
linker+*dddA*	XTEN linker [27]	*gacA-dddA* 3	*gacA-dddA* 4
gacA 3' flank	PAO1 gDNA	*gacA-dddA* 5	*gacA-dddA* 6

pEXG2-*fleQ-dddA*

Fragment	Template	F primer	R primer
pEXG2/HindIII/XhoI	NA		
3' end of *fleQ*	PAO1 gDNA	*fleQ-dddA* 1	*fleQ-dddA* 2
linker+*dddA*	XTEN linker [27]	*fleQ-dddA* 3	*fleQ-dddA* 4
fleQ 3' flank	PAO1 gDNA	*fleQ-dddA* 5	*fleQ-dddA* 6

pUC18T-miniTn7T-Gm-p_{BAD}-araE-*dddAI*

Fragment	Template	F primer	R primer
pUC18T-miniTn7T-Gm-pBAD-*araE*/KpnI/HindIII	NA		
dddA_I	*ddd*A_I [24]	pUC18-*ddd*A_I-F	pUC18-*ddd*A_I-R

pUC18T-miniTn7T-Gm-p_{BAD}-araE-*dddAI*-FLAG

Fragment	Template	F primer	R primer
pUC18T-miniTn7T-Gm-pBAD-araE/KpnI/HindIII	NA		
dddA_I-FLAG	*ddd*A_I [24]	pUC18-*ddd*A_I-F	pUC18-*ddd*A_I-FLAG-R

pPSV39-*ugi*

Fragment	Template	F primer	R primer
pPSV39/SacI/XbaI	NA		
ugi	ugi gBlock codon optimized	pPSV39-UGI-F	pPSV39-UGI-R

pEXG2-Δ*gcsR*

Fragment	Template	F primer	R primer
pEXG2/HindIII /KpnI	NA		
gcsR 5' flank	PAO1 gDNA	pEX.del.gcsR 1	pEX.del.gcsR 2
gcsR 3' flank	PAO1 gDNA	pEX.del.gcsR 3	pEX.del.gcsR 4

pEXG2-Δ*fleQ*

Fragment	Template	F primer	R primer
pEXG2/HindIII/KpnI	NA		
fleQ 5' flank	PAO1 gDNA	pEXG2.del.fleQ 1	pEXG2.del.fleQ 2
fleQ 3' flank	PAO1 gDNA	pEXG2.del.fleQ 3	pEXG2.del.fleQ 4

pEXG2-*gcvH2::gfp-mut3*

Fragment	Template	F primer	R primer
pEXG2/HindIII/KpnI	NA		
gcvH2 5' flank	PAO1 gDNA	MJG2312	MJG2313
gfp-mut3	RP1868	MJG2314	MJG2315
gcvH2 3' flank	PAO1 gDNA	MJG2316	MJG2317

pEXG2-GcsR-V

Fragment	Template	F primer	R primer
pEXG2/HindIII /BamHI	NA		
gacA 5' flank	PAO1 gDNA	pEX-GcsR-V 1	pEX-GcsR-V 2
gacA 3' flank	PAO1 gDNA	pEX-GcsR-V 3	pEX-GcsR-V 4

pEXG2-GacA-V

Fragment	Template	F primer	R primer
pEXG2/HindIII /BamHI	NA		
fleQ 5' flank	PAO1 gDNA	pEX-GacA-V 1	pEX-GacA-V 2
fleQ 3' flank	PAO1 gDNA	pEX-GacA-V 3	pEX-GacA-V 4

pEXG2-FleQ-V

Fragment	Template	F primer	R primer
pEXG2/HindIII /BamHI	NA		
fleQ 5' flank	PAO1 gDNA	pEX-FleQ-V 1	pEX-FleQ-V 2

fleQ 3' flank	PAO1 gDNA	pEX-FleQ-V 3	pEX-FleQ-V 4

pRE112-*ntrC-dddA*

Fragment	Template	F primer	R primer
pRE112/SacI/XbaI	NA		
3' end of *ntrC*	MG1655 gDNA	*ntrC-dddA* 1	*ntrC-dddA* 2
linker+*dddA*	XTEN linker [27]	*ntrC-dddA* 3	*ntrC-dddA* 4
ntrC 3' flank	MG1655 gDNA	*ntrC-dddA* 5	*ntrC-dddA* 6

pMMB67EH-$dddA_I$

Fragment	Template	F primer	R primer
pMMB67EH/ EcoRI/BamHI	NA		
$dddA_I$	$dddA_I$[24]	pMMB-dddI-F	pMMB-dddI-R

pMMB67EH-$dddA_I$-FLAG

Fragment	Template	F primer	R primer
pMMB67EH/ EcoRI/BamHI	NA		
$dddA_I$	$dddA_I$[24]	pMMB-dddI-F	pMMB-dddI-FLAG-R

pPSV39-$dddA_I$-FLAG

Fragment	Template	F primer	R primer
pPSV39/SacI/XbaI	NA		
$dddA_I$	$dddA_I$[24]	pPSV-dddI-FLAG_F	pPSV-dddI-FLAG_R

pBS10-riboE-$dddA_I$

Fragment	Template	F primer	R primer
pBS10//	NA		
$dddA_I$	$dddA_I$[24]	pBS10-riboE-dddI-F	pBS10-dddI-R

pBS10-riboE-$dddA_I$-FLAG

Fragment	Template	F primer	R primer
pBS10/KpnI/SacI	NA		
$dddA_I$	$dddA_I$-FLAG[24]	pBS10-riboE-dddI-F	pBS10-dddI-FLAG-R

pEPSA5-dddA

Fragment	Template	F primer	R primer
pEPSA5/KpnI/BamHI	NA		
dddA	pSCrhaB2-*dddA*	pEPSA5-	pEPSA5-dddA-R

dddA-F

Supplementary Table 7. Primer sequences

Primer	Sequence
pSCRhaB::dddAI-F	TGAAATTCAGCAGGATCACATATGTACGCAGACGATTTCGAC
pSCRhaB::dddAI-R	TCATTTCAATATCTGTATATCTAGATTACAACTCGCTCCATGTC
gcsR-dddA 1	GGAAGCATAAATGTAAAGCAAGCTTGCAACCTGGAGAAGATG GTCGCCG
gcsR-dddA 2	TACCTCCAGAGGCGCGGACCGATGCC
gcsR-dddA 3	TCCGCGCGCCTCTGGAGGTAGCTCCGGC
gcsR-dddA 4	GCCCGCTTCAACAACCTCCTTTCGTGGG
gcsR-dddA 5	AGGAGGTTGTTGAAGCGGGCTCAGCCCT
gcsR-dddA 6	TTAAGGTACCGAATTCGAGCTCGAGCAATCCCAAGGAGTTCG AGCG
gacA-dddA 1	GGAAGCATAAATGTAAAGCAAGCTTCGGATGTCGTCCTGATG GAC
gacA-dddA 2	TACCTCCAGAGCTGGCGGCATCGACCAT
gacA-dddA 3	TGCCGCCAGCTCTGGAGGTAGCTCCGGC
gacA-dddA 4	CGCTCATCTAACAACCTCCTTTCGTGGG
gacA-dddA 5	AGGAGGTTGTTAGATGAGCGCCGTTTTCGACGC
gacA-dddA 6	TTAAGGTACCGAATTCGAGCTCGAGGGCCGCGTACGGTTGCG G
fleQ-dddA 1	GGAAGCATAAATGTAAAGCAAGCTTTCGCCCTGCTGCTCAACG
fleQ-dddA 2	TACCTCCAGAATCATCCGACAGGTCGTCG
fleQ-dddA 3	GTCGGATGATTCTGGAGGTAGCTCCGGC
fleQ-dddA 4	CGACCTGTCAACAACCTCCTTTCGTGGG
fleQ-dddA 5	AGGAGGTTGTTGACAGGTCGTTTCGCAACGCTTTG
fleQ-dddA 6	TTAAGGTACCGAATTCGAGCTCGAGCGCGCGGAGCGAAGCAG C
pPSV39-UGI-F	GATAACAATTTCAGAATTCGAGCTCACGGGAGGAAAGATGAC GAATCTCAGCGACAT
pPSV39-UGI-R	TCATTTCAATATCTGTATATCTAGATTAGAGCATCTTGATTTTG TTCTCGC
pUC18-dddAI-F	GGGCTAGCGAATTCGAGCTCGGTACCACGGGAGGAAAGATGT AC
pUC18-dddAI-R	CTCATCCGCCAAAACAGCCAAGCTTTCACAACTCGCTCCATGT C
pUC18-dddAI-FLAG-R	CTTCTCTCATCCGCCAAAACAGCCAAGCTTTCATTTGTCGTCGT CGTCTTTGTAGTCCAACT CGCTCCATGTCAG
pEX.del.gcsR 1	CATAAATGTAAAGCAAGCTTGGTACCGAGGCGGACT
pEX.del.gcsR 2	AGCCCGCTTCAGGCGCGCGGGATGCGCATGCGGGA
pEX.del.gcsR 3	CAGGTTCCCGCATGCGCATCCCGCGCGCCTGAAGC
pEX.del.gcsR 4	TCGAGCTCGAGCCCGGGGATCCTTCGATTACCCACCTGC
pEXG2.del.fleQ 1	CATAAATGTAAAGCAAGCTTACGCAACAACTGGATGAA

pEXG2.del.fleQ 2	ACGACCTGTCAATCATCCGAGCGCCACATTTTGATCA
pEXG2.del.fleQ 3	AGCTGATCAAAATGTGGCGCTCGGATGATTGACAGGTC
pEXG2.del.fleQ 4	TCGAGCTCGAGCCCGGGGATCCGCAGGGAAATCTCGTGA
MJG2312	GGAAGCATAAATGTAAAGCAAGCTTTTCCTGCTCGCCCACCTG
MJG2313	AAAAGTTCTTCTCCTTTACTCAACTTGCTCATGCTGGATCCTCAT
MJG2314	CCAGCATGAGCAAGTTGAGTAAAGGAGAAGAACTTTTCACTGGAGTTGTC
MJG2315	GTCATGACGGGATCCTCATTTGTATAGTTCATCCATGCCATGTGTAATCC
MJG2316	TGGCATGGATGAACTATACAAATGAGGATCCCGTCATGACCGATAAC
MJG2317	GTGGAAATTAATTAAGGTACCCTGGTGCGGTTCTTCGACAGG
pEX-GcsR-V 1	CATAAATGTAAAGCAAGCTTACCTGTTCTACCGCCTCA
pEX-GcsR-V 2	CTTGCCGAGGCGGTTCATTTCGATGTCGGTGTAAGCGGCCGCGGCGCGCGGACCGATGC
pEX-GcsR-V 3	GCGGCCGCTTACACCGACATCGAAATGAACCGCCTCGGCAAGTGAAGCGGGCTCAGCCC
pEX-GcsR-V 4	TCGAGCTCGAGCCCGGGGATCCGAGTTCGAGCGCTTCAG
pEX-GacA-V 1	CATAAATGTAAAGCAAGCTTGAACTGAAGCCGGATGTC
pEX-GacA-V 2	CTTGCCGAGGCGGTTCATTTCGATGTCGGTGTAAGCGGCCGCGCTGGCGGCATCGACCA
pEX-GacA-V 3	GCGGCCGCTTACACCGACATCGAAATGAACCGCCTCGGCAAGTAGATGAGCGCCGTTTTC
pEX-GacA-V 4	TCGAGCTCGAGCCCGGGGATCCGCGCTCGGATAGGGACC
pEX-FleQ-V 1	GCATAAATGTAAAGCAAGCTTCTGCTGCTCAACGAACTG
pEX-FleQ-V 2	CTTGCCGAGGCGGTTCATTTCGATGTCGGTGTAAGCGGCCGCATCATCCGACAGGTCGTC
pEX-FleQ-V 3	GCGGCCGCTTACACCGACATCGAAATGAACCGCCTCGGCAAGTGACAGGTCGTTTCGCAA
pEX-FleQ-V 4	TCGAGCTCGAGCCCGGGGATCCCGCCAGAGCATGCCCACC
ung_del-F	TAGAAAGAAGCAGTTAAGCTAGGCGGATTGAAGATTCGCAGGAGAGCGAGATGGTGTAGGCTGGAGCTGCTTC
ung_del-R	TGATAAATCAGCCGGGTGGCAACTCTGCCATCCGGCATTTCCCCGCAAATTTACATATGAATATCCTCCTTAG
ntrC-dddA 1	CATGAATTCCCGGGAGAGCTATTCCTCGACGAAATTGGTG
ntrC-dddA 2	TACCTCCAGACTCCATCCCCAGCTCTTTTAAC
ntrC-dddA 3	GATGGAGTCTGGAGGTAGCTCCGGC
ntrC-dddA 4	GCTGTGAACTAACAACCTCCTTTCGTGGG
ntrC-dddA 5	AGGTTGTTAGTTCACAGCTTGTGTGTAAAG
ntrC-dddA 6	GGCCCGATCCCAAGCTTCTTTCATCTGCAACTTCCGTC
PA2869 qPCR F	GGCGCTGAACGACTAC
PA2869 qPCR R	GGCGATAGGCGAGGATG
2870 qPCR F1	GCTGCTCGATCCGACAAA

2870 qPCR R1	GATCAGCGCCATGAGCA
pelA qPCR F	CCGAGCTTTCCCAGTTC
pelA qPCR R	CTGCCCTGCTCTTTCAG
yfiR qPCR F	CTTCGTTGCCCACCTTG
yfiR qPCR R	TATGCCCAGCAGAACCT
PA3340 qPCR F	CGGAATGCCCGGTAAAG
PA3340 qPCR R	GATGTCTCCCACTCCCA
pGcsR-F	TATCGGTCATGACGGGATCCTC
pGcsR-R	TGGTCTTCCAGCACCCTTCG
pGcsR-seq	TGTTGCTGGCGGCCTT
pMMB-dddI-F	CAATTTCACACAGGAAACAGAATTCACGGGAGGAAAGATGTAC
pMMB-dddI-R	CCTGCAGGTCGACTCTAGAGGATCCTCACAACTCGCTCCATGTC
pMMB-dddI-FLAG-R	CAGGTCGACTCTAGAGGATCCTCATTTGTCGTCGTCGT
pPSV-dddI-FLAG-F	AATTTCAGAATTCGAGCTCACGGGAGGAAAGATGTACGCAGACGATTTCG
pPSV-dddI-FLAG-R	TCATTTCAATATCTGTATATTCTAGATCATTTGTCGTCGTCGTC
pBS10-riboE-dddI-F	ATACGACTCACTATAGGTACCGGTGATACCAGCATCGTCTTGATGCCCTTGGCAGCACCCTGCTAAGGAGGTAACAACAAGATGATGTACGCAGACGATTTCG
pBS10-dddI-R	GCTTTGTTAGCAGCCGAGCTCTCACAACTCGCTCCATGTC
pBS10-dddI-FLAG-R	GCTTTGTTAGCAGCCGAGCTCTCATTTGTCGTCGTCGTC
pEPSA5-dddA-F	GTAGAATTCGAGCTCGGTACCAAATAGAAGGATGATGAAAATGATAGGACTCAACGGTG
pEPSA5-dddA-R	GCTGCAGGTCGACTCTAGAGGATCCTCATTTTCCTAATCTATTCATTTCAATATC

References

1 Brown, D. R., Barton, G., Pan, Z., Buck, M. & Wigneshweraraj, S. Nitrogen stress response and stringent response are coupled in Escherichia coli. *Nat Commun* **5**, 4115, doi:10.1038/ncomms5115 (2014).

2 Aquino, P. *et al.* Coordinated regulation of acid resistance in Escherichia coli. *BMC Syst Biol* **11**, 1, doi:10.1186/s12918-016-0376-y (2017).

3 Landt, S. G. *et al.* ChIP-seq guidelines and practices of the ENCODE and modENCODE consortia. *Genome Res* **22**, 1813-1831, doi:10.1101/gr.136184.111 (2012).

4 Stover, C. K. *et al.* Complete genome sequence of Pseudomonas aeruginosa PA01, an opportunistic pathogen. *Nature* **406**, 959–964 (2000).

5 de Moraes, M. H. *et al.* An interbacterial DNA deaminase toxin directly mutagenizes surviving target populations. *Elife* **10**, doi:10.7554/eLife.62967 (2021).

6 Boyer, H. W. & Roulland-Dussoix, D. A complementation analysis of the restriction and modification of DNA in Escherichia coli. *J Mol Biol* **41**, 459-472, doi:10.1016/0022-2836(69)90288-5 (1969).

7 Simon, R., Priefer, U. & Puher, A. A Broad Host Range Mobilization System for In Vivo Genetic Engineering: Transposon Mutagenesis in Gram Negative Bacteria. *Nat. Biotechnol.* **1**, 784–791 (1983).

8 Monk, I. R., Tree, J. J., Howden, B. P., Stinear, T. P. & Foster, T. J. Complete Bypass of Restriction Systems for Major Staphylococcus aureus Lineages. *MBio* **6**, e00308-00315, doi:10.1128/mBio.00308-15 (2015).

9 Fey, P. D. *et al.* A genetic resource for rapid and comprehensive phenotype screening of nonessential Staphylococcus aureus genes. *MBio* **4**, e00537-00512, doi:10.1128/mBio.00537-12 (2013).

10 Rietsch, A., Vallet-Gely, I., Dove, S. L. & Mekalanos, J. J. ExsE, a secreted regulator of type III secretion genes in Pseudomonas aeruginosa. *Proc Natl Acad Sci U S A* **102**, 8006–8011 (2005).

11 Cardona, S. T. & Valvano, M. A. An expression vector containing a rhamnose-inducible promoter provides tightly regulated gene expression in Burkholderia cenocepacia. *Plasmid* **54**, 219–228 (2005).

12 Silverman, J. M. *et al.* Haemolysin Coregulated Protein Is an Exported Receptor and Chaperone of Type VI Secretion Substrates. *Molecular Cell* **51**, 584–593, doi:10.1016/j.molcel.2013.07.025 (2013).

13 Kulasekara, B. R. *et al.* c-di-GMP heterogeneity is generated by the chemotaxis machinery to regulate flagellar motility. *Elife* **2**, e01402, doi:10.7554/eLife.01402 (2013).

14 Figurski, D. H. & Helinski, D. R. Replication of an origin-containing derivative of plasmid RK2 dependent on a plasmid function provided in trans. *Proc Natl Acad Sci U S A* **76**, 1648-1652, doi:10.1073/pnas.76.4.1648 (1979).

15 Choi, K. H. *et al.* Genetic tools for select-agent-compliant manipulation of Burkholderia pseudomallei. *Applied and environmental microbiology* **74**, 1064–1075 (2008).

16 Hoang, T. T., Karkhoff-Schweizer, R. R., Kutchma, A. J. & Schweizer, H. P. A broad-host-range Flp-FRT recombination system for site-specific excision of chromosomally-located DNA sequences: application for isolation of unmarked

Pseudomonas aeruginosa mutants. *Gene* **212**, 77-86, doi:10.1016/s0378-1119(98)00130-9 (1998).

17 Furste, J. P. *et al.* Molecular cloning of the plasmid RP4 primase region in a multi-host-range tacP expression vector. *Gene* **48**, 119-131, doi:10.1016/0378-1119(86)90358-6 (1986).

18 Corrigan, R. M. & Foster, T. J. An improved tetracycline-inducible expression vector for Staphylococcus aureus. *Plasmid* **61**, 126-129, doi:10.1016/j.plasmid.2008.10.001 (2009).

19 Santiago, M. *et al.* A new platform for ultra-high density Staphylococcus aureus transposon libraries. *BMC genomics* **16**, 252, doi:10.1186/s12864-015-1361-3 (2015).

20 Edwards, R. A., Keller, L. H. & Schifferli, D. M. Improved allelic exchange vectors and their use to analyze 987P fimbria gene expression. *Gene* **207**, 149–157 (1998).

21 Datsenko, K. A. & Wanner, B. L. One-step inactivation of chromosomal genes in Escherichia coli K-12 using PCR products. *Proc Natl Acad Sci U S A* **97**, 6640–6645, doi:10.1073/pnas.120163297 (2000).

22 Mougous, J. D. *et al.* A virulence locus of Pseudomonas aeruginosa encodes a protein secretion apparatus. *Science* **312**, 1526–1530 (2006).

23 LeRoux, M. *et al.* Kin cell lysis is a danger signal that activates antibacterial pathways of Pseudomonas aeruginosa. *Elife* **4**, doi:10.7554/eLife.05701 (2015).

24 Mok, B. Y. *et al.* A bacterial cytidine deaminase toxin enables CRISPR-free mitochondrial base editing. *Nature* **583**, 631-637, doi:10.1038/s41586-020-2477-4 (2020).

25 Wirth, R., An, F. Y. & Clewell, D. B. Highly efficient protoplast transformation system for Streptococcus faecalis and a new Escherichia coli-S. faecalis shuttle vector. *J Bacteriol* **165**, 831-836, doi:10.1128/jb.165.3.831-836.1986 (1986).

26 Forsyth, R. A. *et al.* A genome-wide strategy for the identification of essential genes in Staphylococcus aureus. *Molecular microbiology* **43**, 1387–1400 (2002).

27 Schellenberger, V. *et al.* A recombinant polypeptide extends the in vivo half-life of peptides and proteins in a tunable manner. *Nat Biotechnol* **27**, 1186-1190, doi:10.1038/nbt.1588 (2009).

Supplementary Note 1
(Statistical analyses of 3D-seq data)

I. STATISTICAL ANALYSIS

We divide the analysis into two steps: peak detection and peak-parameter inference. In the first peak-detection step, we used a canonical frequentist approach: null hypothesis testing [1] to determine the number and approximate position of the peaks in the data. Then, in a second step, we optimized the model parameters describing each peak individually using a slower but more accurate numerical Maximum Likelihood Estimation (MLE) to optimize peak parameter inference.

Before developing the statistical approach from a formal perspective, it is useful to discuss the approach to peak detection qualitatively. The data shown in Fig. 1 is representative of our 3D-seq data. It is useful to think of the allele frequency being generated by two distinct mechanisms: (i) There is a *background* generated by non-localized DddA fusions, which is nearly uniformly distributed over the genome (but stochastic). (ii) When the DddA fusion is localized at a particular genetic locus, its activity is predominantly localized to a *peak* 2-5 kb in width.

The allele frequency at any single site, shown in green in Fig. 1, is very noisy; however, this data can be binned, shown in blue in Fig. 1, to generate a higher allele-frequency signal-to-noise ratio in exchange for lower genomic-position resolution.

To understand the statistical evidence for a peak and the dynamic range of the 3D-seq approach, it is *insufficient* to consider enzyme activity at a single putative conversion site. Rather the statistical evidence and dynamic range arise due to the compounding effect of low activity at hundreds of proximal putative conversion sites surrounding the putative binding site of the fused DNA biding protein. The sensitivity of 3D-seq will depend on a number of factors, but most importantly: the conversion efficiency in proximity to the site of interest, the background DddA enzyme activity and the genome size, the number of putative conversion sites in the vicinity of the putative binding site, *etc*. A careful and quantitative approach to the statistics is therefore necessary.

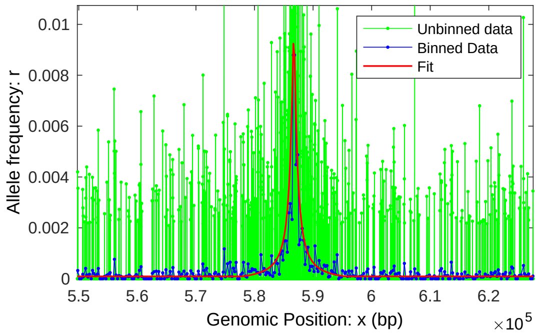

FIG. 1. **A typical fit to a peak.** The unbinned and binned data are compared to the model for the allele frequency for GacA. Although the raw allele frequency appears qualitatively higher than the binned and model frequencies, all three are in fact mutually consistent. The apparent high allele frequencies seen in the raw data are an artifact of the noise in a signal consisting of fluctuations between high allele-frequency values (rare) and zeros (frequent). This noise at the base-pair resolution is a consequence of low DddA expression and the finite population size. In contrast both the binned and model allele frequencies can be understood as averaged to reduce this noise: The binned frequency is averaged over a narrow window of genetic loci whereas the model represents the estimated cell-mean allele frequency (i.e. averaged over an infinite-sized population).

II. BIOPHYSICAL MODEL FOR THE ALLELE FREQUENCY

Motivated by the DNA effective-concentration model (*e.g.* [2]), we modeled the cell-mean allele frequency at locus j as:

$$\overline{r}_j = \mu_0 + \sum_J \delta\mu_J(x_j), \tag{1}$$

where the first term represents the activity on non-localized DddA-transcription-factor fusions and the second term represents the activity, at genomic position x_j, for a fusions specifically bound at binding site J at genomic position ℓ_J. $\delta\mu_J$ will form an allele-frequency peak around site J: it will be large when sequence j and site J are proximal and nearly zero for sequences distal to the sites.

For the functional form of the peak profile, we will again consider the DNA effective-concentration model (*e.g.* [2]). We will model the peak profile as a generalized Cauchy function:

$$C(x/L; a, D) \equiv \left[1 + |x/L|^D\right]^{-a/D}, \tag{2}$$

for $D = 1$. In this model $x \equiv x_j - \ell_J$ is the genomic displacement (in bp) between sequence j and binding site J. The scaling exponent a is a model parameter that controls the rapidity with which the tails decay away from the peak. Its value is determined by chromatin structure and we expect $1 < a < 1.5$ [2–4]. The parameter L defines the width of the peak and depends both on the structure of the protein fusion as well as chromatin structure. The peak profile is shown in Fig. 2.

In practice, it will be convenient to only consider peak profile functions with local position support. We will

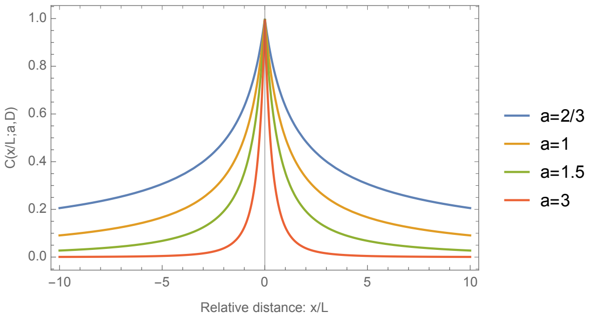

FIG. 2. **The peak profile is modeled by a generalized Cauchy function.** Parameter a controls the power-law decay of the tails with relative genomic distance and parameter D control the geometry of the summit (not shown).

therefore use a generalized Cauchy that is cut off at $|x/L| > 16$:

$$C'(\tfrac{x}{L}; a, D) \equiv \begin{cases} \frac{C(\frac{x}{L};a,D)-C(16;a,D)}{C(0;a,D)-C(16;a,D)}, & |x/L| \le 16 \\ 0, & |x/L| > 16 \end{cases}, \quad (3)$$

which makes no qualitative difference to shape of the peak profile.

Our model for the mean allele frequency at locus j due to specific binding at site J is therefore:

$$\delta\mu_J(x_j) \equiv \delta\mu(x_j; \theta_J) \equiv I_J C'(\tfrac{x_j-\ell_J}{L_J}; a_J, 1), \quad (4)$$

where the parameter vector contains the following parameters:

$$\theta_J = (I_J, \ell_J, a_J, L_J), \quad (5)$$

and the last undefined parameter I_J controls the peak profile amplitude (or height). The model results in an excellent fit to the observed allele frequency peaks, as shown in Fig. 1.

1. *Modeling of the distribution of allele frequency*

Our model so far describes the cell-mean allele frequency at a genetic locus, however the creation of alleles is a stochastic process. Furthermore, the cells are mutated while the culture is growing, therefore alleles that are created early, grow with the population and have a higher frequency than alleles created late in the growth process. This is the well-known *jackpot phenomenon* [5]. Although this type of principled analysis is possible it would require a great number of parameters and potentially experimental calibrations [6]. Instead, we will implement a much more tractable and practical approach to the modeling of the expected distribution of allele frequencies at a given locus: We will model r_i as a Gaussian random variable with a locus-specific mean (Eq. 1) and variance that is proportional to the mean.

Let the data D be defined as the set of N allele frequencies r_i and genomic positions x_i pairs:

$$D \equiv \{(x_i, r_i)\}_{i=1\ldots N}. \quad (6)$$

We modeled the allele frequencies r_i as a Gaussian random variable. We assume a *locus-dependent* mean μ_i and variance σ_i^2:

$$R_i \sim \mathcal{N}(\mu_i, \sigma_i), \quad (7)$$

where R_i is capitalized because it is being interpreted as a random variable, $\mathcal{N}$ is the normal distribution.

III. PEAK DETECTION BY NULL HYPOTHESIS TESTING

1. *Data binning and processing for peak detection*

Since the peak features are wide compared to single basepair resolution, it is convenient to detect the peaks initially at low resolution before optimizing the peak parameters using the full resolution data. The central limit theorem guarantees that for sufficiently large bins, the $r_{j'}$ will be normally distributed, simplifying the analysis. However, as the size of the bins grows, so does the noise from the background enzyme activity. We compromised with a bin size of 250 bp.

One important feature of the allele frequency data is that not every base is a target due to the TC-sequence specificity of DddA. We therefore binned the data using a protocol that avoided the introduction of bins weighted by the number of target sites. We divided the genome into 250 bp bins. In each bin j', we have data indexes $j \in \mathcal{J}_{j'}$. We defined the position of the binned target $x_{j'}$ as the weighted average of the sites in that bin:

$$x_{j'} \equiv \frac{\sum_{j\in\mathcal{J}_J} x_j r_j}{\sum_{j\in\mathcal{J}_J} r_j}. \quad (8)$$

If all r_j were zero in the bin, the index position $x_{j'}$ was assigned the mean of x_j for $j \in \mathcal{J}_{j'}$. If no positions existed in the bin, the bin was omitted from the analysis. The allele frequency for the binned data $r_{j'}$ was equal to the mean of the r_j for $j \in \mathcal{J}_{j'}$. The binned data is shown in Fig. 3.

We found that at 250 bp bin size, there was still a significant amount of salt-and-pepper noise: *i.e.* extremely high allele frequency at single isolated position, surrounded by background level activity. Presumably the source of this noise are jackpot events early in proliferation.

To eliminate the jackpot features we use a standard approach from image processing [7]: We generated median filtered allele frequency $\tilde{r}_{j'}$ by taking a median of $r_{j'}$ using the neighborhood $[j'-1, j', j'+1]$. If $r_{j'}$ was four standard deviations above the median-filter value

$\tilde{r}_{j'}$, we replace $r_{j'}$ with the median-filtered value $\tilde{r}_{j'}$. The binned and filtered data is shown in Fig. 3.

Note that this binned data $D' = \{(x_{j'}, r_{j'})\}$ is used only in peak detection and the raw (unbinned and unfilter) data $D = \{(x_i, r_i)\}$ is used for model parameter refinement.

2. Implementing the locus-dependent variance in the test statistic

Since the dataset is dominated by peak-free regions, we can write:

$$\sigma_i^2 \approx \sigma_0^2 \frac{\mu_i}{\mu_0}, \tag{9}$$

where μ_0 are σ_0^2 and mean and variance over the entire dataset and μ_i is the locus-dependent mean (Eq. 1). In this case we know that the tails of the distribution away from the peaks peaks look exponential and not Gaussian. (See Fig. 3B.) If we force the likelihood to be Gaussian, this will inflate the p values. It is therefore convenient to implement the variance model in the following way:

$$\sigma_i^2 \to \sigma_0^2 \max(1, r_i/\mu_0). \tag{10}$$

If the $r_i \gg \mu 0$, the term in the exponent will now be linear:

$$\tfrac{1}{2\sigma_i^2}(r_i - \mu_0)^2 \approx \tfrac{1}{2\sigma_0^2} r_i \mu_0, \tag{11}$$

rather than quadratic:

$$\tfrac{1}{2\sigma_0^2}(r_i - \mu_0)^2 \approx \tfrac{1}{2\sigma_0^2} r_i^2. \tag{12}$$

This linear dependence matches the observed distribution which decays exponentially in r. (See Fig. 3B.) We will use this approach for estimating the p value using the binned data for peak detection.

Another approach will be implemented for parameter approximation (Sec. IV.)

3. Parameter maximum likelihood estimation for peak detection

The first step in the null hypothesis test is to perform a maximum likelihood estimate (MLE) of the parameter values. Since the peaks constitute a negligible fraction of the sequence, we will estimate the background mean μ_0 and variance σ_0^2 using the MLE analysis in the null hypothesis and leave these fixed in all nested models. In what follows, parameters will refer only to the parameters describing the peak profiles. Each peak J will be described by θ_J.

To estimate the parameters from the peak profile, we must first write the minus-log-likelihood for the normal model at the N positions:

$$-\log q(D|\theta) = \tfrac{N}{2} \log 2\pi\sigma_i^2 + \textstyle\sum_{i=1}^{N} \tfrac{1}{2\sigma_i^2}[r_i - \mu_i]^2, \tag{13}$$

where the position-dependent mean $\mu_i(\theta)$ depends on the model through the peak profile function (Eqs. 1-4) and σ_i^2 is approximated using Eq. 10. We now need to minimize Eq. 13 with respect to the parameters θ.

One difficulty here is that this statistical problem is singular: As $I \to 0$ the peak position ℓ becomes unidentifiable [8]. We therefore must take a brute-force approach to estimating ℓ. We use the following steps: (i) We considered a reduced sets of positions $\ell \in \mathfrak{X} \equiv \{x_i\}_{i=1...N}$. We exhaustively consider a peak position at each $\ell = x_i$. (ii) The parameters L and a were fairly consistent between peaks since they are determined by gross-level chromatin structure. Therefore in the process of peak *detection*, we will assign all peak the global parameter values $\hat{L} \to 400$ bp and $\hat{a} \to 1.5$. (iii) The final unknown MLE parameter $\hat{I}$ can be estimated easily since $\delta\mu_i \propto I$ and a closed-form expression can be derived for it. Since C' has only local support, the MLE estimates can be computed rapidly.

4. Null hypothesis testing

The test statistic λ in the likelihood ratio test is

$$\lambda(D) \equiv \log \frac{q_1(D|\hat{\theta})}{q_0(D)}. \tag{14}$$

We use the canonical Neyman-Pearson approach to hypothesis testing [1]. We chose a confidence level of $\gamma = 95\%$ (*i.e.* a significance level of 5%). The peak exists, *i.e.* we will reject the null hypothesis, if

$$F_\Lambda(\lambda) > \gamma, \tag{15}$$

where F_Λ is the Cumulative Distribution Function (CDF) of the test statistic λ under the null hypothesis. (Note that λ is capitalized because it is being interpreted as a random variable.)

5. Bootstrap estimate of F_Λ

Under the normal course of events, if the model were regular in the large sample size limit, we could use the Wilk's theorem to relate the distribution of Λ under the null hypothesis to a chi-squares distribution [9]. However, the model is singular [8] and we must therefore estimate the distribution of the test statistic explicitly.

To compute the distribution of the test statistic, we use a stochastic simulation of the null hypothesis and then compute the empirical distribution of the test statistic. Initially we attempted to use a Gaussian random variable to simulate the null hypothesis data, however the estimated p values were too small. In retrospect, it is pretty clear from Fig. 3B that the r-distribution tails decay exponentially and therefore large r_i's are much more frequent than predicted by a Gaussian distribution.

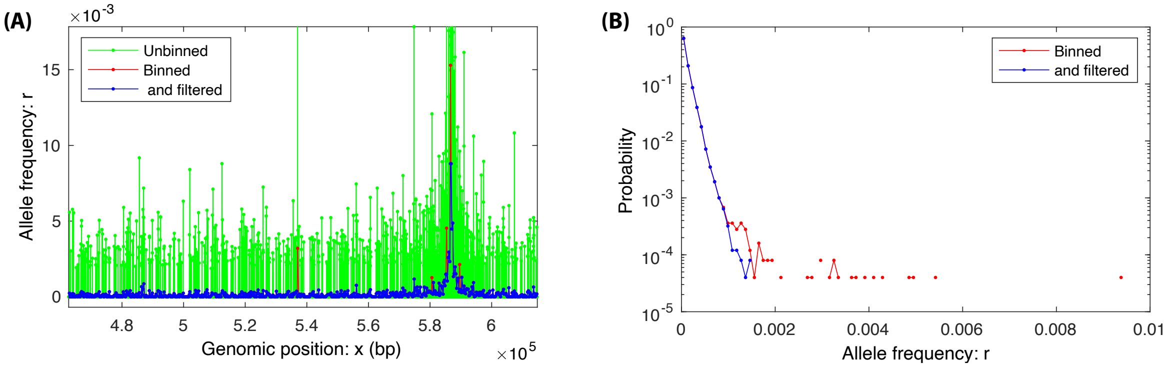

FIG. 3. **Data processing for peak detection for GacA. Panel A:** The raw data (green) was first binned to 250 bp bins (red). Salt-and-pepper noise, isolated high values, were then removed to generated filtered data (blue). Peak detection was performed on the binned and filtered data (blue). Final parameter fits were performed on unbinned and unfiltered data (green). **Panel B:** A histogram of allele frequencies r_i. Very few allele frequencies were removed by filtering.

In this situation, one can uses a bootstrap method to estimate the test statistic [10]. There are two tractable choices: (i) a the canonical bootstrap approach samples from the empirical distribution consisting of the finite set of observed background allele frequencies shown in Fig. 3B. (ii) A parametric bootstrap method fits the observed distribution to an empirical model and then uses the model to generate simulated data. We used the parametric bootstrap since it had the ability to sample even-more-extreme allele frequencies than were observed. We fit the the distribution of Allele frequencies r_i for the background for the GacA data to the empirical model for random variable R:

$$\begin{aligned} p_R(r) &= P_0 \cdot \delta(r) + ... \\ &+ \Theta(r)\Theta(r_0 - r) \cdot (1 - P_0)\tfrac{b_1 b_2}{b_+ b_2} e^{-b_1(r_0 - r)} + ... \\ &+ \Theta(r - r_0) \cdot (1 - P_0)\tfrac{b_1 b_2}{b_+ b_2} e^{-b_2(r - r_0)}, \end{aligned} \quad (16)$$

where δ and Θ are the Dirac delta and Heaviside function respectively. See Fig. 4. The empirical model parameters were fit using an MLE approach:

$$r_0 = 7.2906 \times 10^{-5}, \quad (17)$$
$$P_0 = 5.386 \times 10^{-1}, \quad (18)$$
$$b_1 = 1.2972 \times 10^{5}, \quad (19)$$
$$b_2 = 8.4846 \times 10^{3}. \quad (20)$$

The fit of the empirical model to the background allele frequency is excellent and is shown in Fig. 4.

6. *Estimating the distribution of the test statistic*

Using the parametric-bootstrap model, we simulated the null hypothesis data $D' = \{(x_{j'}, R_{j'})\}$ where $R_{j'} \sim p_R$. For each simulated dataset, we then computed the test statistic:

$$\Lambda \equiv \lambda(D'), \quad (21)$$

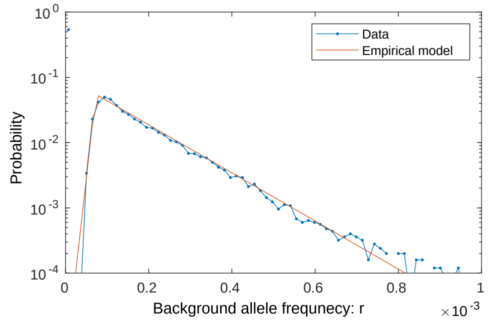

FIG. 4. **Empirical model for parametric bootstrap.** The measured background allele frequency is shown in blue and the empirical model fit by MLE analysis is shown in red.

which we interpret as a random variable. We generated 10^5 samples of Λ. We then use the empirical distribution of Λ to estimate the p values in the usual way (e.g. [10]).

7. *Computation of the p value*

We have included a p value for each detected peak as a proxy for statistical support. The p value for test statistic λ is:

$$p(\lambda) = 1 - F_\Lambda(\lambda). \quad (22)$$

Since some of peaks are extremely large, the observed test statistic is much larger than any observed in our simulations. To estimate the p values in this context, we fitted the empirical distribution F_Λ to a Gumbel distribution since the minimization of the minus-log-likelihood over ℓ can be reinterpreted as an extreme value problem for a random variable in the exponential

family [11]. The Gumbel distribution is

$$F_\Lambda(\lambda) = e^{-e^{-\frac{\lambda-\mu_\Lambda}{\sigma_\Lambda}}}, \tag{23}$$

where the position and scale parameters are

$$\mu_\Lambda = 0.7217, \tag{24}$$
$$\sigma_\Lambda = 0.2823, \tag{25}$$

respectively, which we estimated using an MLE approach. For very small p we can make the following approximation:

$$\log p \approx -\tfrac{\lambda-\mu_\lambda}{\sigma_\lambda}, \tag{26}$$

by Taylor expanding the outer-most exponential around zero in Eq. 23.

8. *Statistical tests for subsequent nested models*

After a peak is detected by rejecting the null hypothesis, we replace the null hypothesis with the alternative hypothesis and then define a new alternative hypothesis with another putative peak. We then repeat the null hypothesis test. This procedure was repeated until no more statistically significant peaks could be detected.

IV. PARAMETER INFERENCE AND FIT REFINEMENT

Once the peaks were detected, we refined all four profile parameters, $\theta = (I, \ell, a, L)$, for each peak by direct numerical maximum likelihood estimation for all parameters, now all defined on $\mathbb{R}$. Note that this optimization is performed *after* peak detection. This refinement is performed on the full resolution data.

For parameter inference we will use a different approach for the scaling of the variance:

$$\sigma_i^2 = \sigma_0^2 \frac{\mu_i}{\mu_0}, \tag{27}$$

since the approximation in Eq. 10 fails for the higher resolution data. For parameter optimization, the tails of the distribution are of little importance.

To estimate the uncertainty in the parameters, we used the Fisher information in the usual way (e.g. [1]). The numerical minimization resulted in a Jacobian:

$$J_{\alpha i} \equiv [\partial_{\theta_\alpha} \delta\mu_i/\sigma_i,](\hat{\theta}). \tag{28}$$

The Fisher information is then:

$$I = [JJ^T], \tag{29}$$

and therefore the predicted covariance in error is

$$\overline{\delta\theta_\alpha \delta\theta_\beta} = [I^{-1}]_{\alpha\beta}. \tag{30}$$

[1] D. R. Cox and D. V. Hinkley, *Theoretical Statistics* (Chapman & Hall, 1974).

[2] K. Rippe, P. H. von Hippel, and J. Langowski, Trends in Biochemical Sciences **20**, 500 (1995).

[3] J. Dekker, K. Rippe, M. Dekker, and N. Kleckner, Science **295**, 1306 (2002).

[4] E. Lieberman-Aiden, N. L. van Berkum, L. Williams, M. Imakaev, T. Ragoczy, A. Telling, I. Amit, B. R. Lajoie, P. J. Sabo, M. O. Dorschner, R. Sandstrom, B. Bernstein, M. A. Bender, M. Groudine, A. Gnirke, J. Stamatoyannopoulos, L. A. Mirny, E. S. Lander, and J. Dekker, Science **326**, 289 (2009).

[5] S. E. Luria and M. Delbrück, Genetics **28**, 491 (1943).

[6] Q. Zheng, Mathematical Biosciences **162**, 1 (2010).

[7] R. C. Gonzalez and R. E. Woods, *Digital image processing* (Prentice Hall, Upper Saddle River, N.J., 2008).

[8] S. Watanabe, Journal of Machine Learning Research. **14**, 867 (2013).

[9] S. S. Wilks, The Annals of Mathematical Statistics. **9**, 60 (1938).

[10] B. Efron and R. Tibshirani, *An Introduction to the Bootstrap.* (Chapman & Hall/CRC, Boca Raton, FL, 1993).

[11] L. Haan and A. Ferreira, *Extreme value theory: an introduction.* (Springer., 2007).
