# Supplementary material for: Genome-wide protein–DNA interaction site mapping in bacteria using a double-stranded DNA-specific cytosine deaminase
Source: Nat Microbiol. 2022 Jun 1;7(6):844–55. doi: 10.1038/s41564-022-01133-9 (PMC9159945; doi:10.1038/s41564-022-01133-9)
Supplement: Supplementary file 2 — Reporting Summary [file 41564_2022_1133_MOESM2_ESM.pdf]

nature portfolio

Corresponding author(s): Joseph D. Mougous

Last updated by author(s): 22/04/2022

Reporting Summary

Nature Portfolio wishes to improve the reproducibility of the work that we publish. This form provides structure for consistency and transparency in reporting. For further information on Nature Portfolio policies, see our Editorial Policies and the Editorial Policy Checklist.

Statistics

For all statistical analyses, confirm that the following items are present in the figure legend, table legend, main text, or Methods section.

n/a	Confirmed	
☐	☒	The exact sample size (n) for each experimental group/condition, given as a discrete number and unit of measurement
☐	☒	A statement on whether measurements were taken from distinct samples or whether the same sample was measured repeatedly
☐	☒	The statistical test(s) used AND whether they are one- or two-sided<br>*Only common tests should be described solely by name; describe more complex techniques in the Methods section.*
☒	☐	A description of all covariates tested
☐	☒	A description of any assumptions or corrections, such as tests of normality and adjustment for multiple comparisons
☐	☒	A full description of the statistical parameters including central tendency (e.g. means) or other basic estimates (e.g. regression coefficient) AND variation (e.g. standard deviation) or associated estimates of uncertainty (e.g. confidence intervals)
☐	☒	For null hypothesis testing, the test statistic (e.g. F, t, r) with confidence intervals, effect sizes, degrees of freedom and P value noted<br>*Give P values as exact values whenever suitable.*
☒	☐	For Bayesian analysis, information on the choice of priors and Markov chain Monte Carlo settings
☒	☐	For hierarchical and complex designs, identification of the appropriate level for tests and full reporting of outcomes
☒	☐	Estimates of effect sizes (e.g. Cohen's d, Pearson's r), indicating how they were calculated

Our web collection on statistics for biologists contains articles on many of the points above.

Software and code

Policy information about availability of computer code

Data collection	Whole-genome sequencing libraries (3D-seq) and ChIP-seq libraries were prepared as described in methods and sequenced on Illumina NextSeq 550, HiSeq2500 or iSeq instruments using default demultiplexing and filtering settings for generating fastq sequence files.
Data analysis	Commercial and open source software used in data analysis in this study include bowtie2 version 2.3.4.3, QuEST version 2.4, Integrative Genomics Viewer (IGV) version 2.5.0, BEDtools version 2.27.1, HTStream pipeline v. 1.3.0, Minimap2 v. 2.17-r974-dirty, SAMTools v. 1.10 , PySAM v. 0.16.0.1, Biopython v. 1.78, Pandas v. 1.3.0, and MEME V. 5.4.1 . Computer code generated is available from GitHub at https://github.com/marade/3DSeqTools.

For manuscripts utilizing custom algorithms or software that are central to the research but not yet described in published literature, software must be made available to editors and reviewers. We strongly encourage code deposition in a community repository (e.g. GitHub). See the Nature Portfolio guidelines for submitting code & software for further information.

Data

Policy information about availability of data

All manuscripts must include a data availability statement. This statement should provide the following information, where applicable:

- Accession codes, unique identifiers, or web links for publicly available datasets
- A description of any restrictions on data availability
- For clinical datasets or third party data, please ensure that the statement adheres to our policy

Sequence data associated with this study is available from the Sequence Read Archive at BioProject PRJNA748760. Publicly available datasets employed in this study include Pseudomonas aeruginosa PAO1 UW reference sequence (NCBI accession NC002516.2) and the Escherichia coli K-12 MG1655 reference sequence (NCBI accession NC_000913.3).

March 2021

Field-specific reporting

Please select the one below that is the best fit for your research. If you are not sure, read the appropriate sections before making your selection.

☒ Life sciences ☐ Behavioural & social sciences ☐ Ecological, evolutionary & environmental sciences

For a reference copy of the document with all sections, see nature.com/documents/nr-reporting-summary-flat.pdf

Life sciences study design

All studies must disclose on these points even when the disclosure is negative.

Sample size	For 3d-seq experiments, the number of replicate growth cultures (n=4) per strain and condition analyzed was chosen empirically after observing that expected target sites were strongly distinguished from non-target sites by requiring reproducibility of position modification in at least three of four replicates. For ChIP-seq analyses, we employed biological triplicates, which exceeds the duplicates recommended by Landt et al (Genom. Res., 2012). Pre-calculation of sample size was not performed.
Data exclusions	No data were excluded from the analyses.
Replication	For 3D-seq, replicate (n=4) cultures were grown for each experimental condition. For each condition, similar but not identical patterns of enhanced target site modifications were observed among all replicates. For ChIP-seq, triplicate cultures were grown for each regulator examined. Peaks considered significant were those detected in at least 2 of the replicates (among other criteria).
Randomization	Experiments consisted of replicate bacterial cultures of identical genotype (starting from isolated colonies of a single strain) grown under identical conditions (parallel setup and growth). No known covariates were pertinent.
Blinding	Blinding was not possible or relevant because knowing the strain genotypes and culture conditions was integral to performing the experiments.

Reporting for specific materials, systems and methods

We require information from authors about some types of materials, experimental systems and methods used in many studies. Here, indicate whether each material, system or method listed is relevant to your study. If you are not sure if a list item applies to your research, read the appropriate section before selecting a response.

Materials & experimental systems

n/a	Involved in the study	
☐	☒	Antibodies
☒	☐	Eukaryotic cell lines
☒	☐	Palaeontology and archaeology
☒	☐	Animals and other organisms
☒	☐	Human research participants
☒	☐	Clinical data
☒	☐	Dual use research of concern

Methods

n/a	Involved in the study	
☐	☒	ChIP-seq
☒	☐	Flow cytometry
☒	☐	MRI-based neuroimaging

Antibodies

Antibodies used	Anti-VSV-Glycoprotein-Agarose antibody,<br>Mouse monoclonal<br>clone P5D4, purified from hybridoma cell culture, purchased from Sigma (product number A1970).
Validation	From the manufacturer's website: Monoclonal Anti-VSV-Glycoprotein, derived from clone P5D4, recognizes an epitope containing the five carboxy-terminal amino acids of Vesicular stomatitis virus glycoprotein (VSV-G). It recognizes native as well as denatured forms of VSV-G tagged proteins. The product was verified to be active on N-terminal VSV-G tagged fusion proteins expressed in E. coli or in mammalian cells. This Anti-VSV-G antibody has been widely used for the study of cell transport processes. In addition, recombinant proteins tagged with the P5D4 epitope have been detected, immunoprecipitated and localized with the antibody. Monoclonal Anti-VSV-G-Agarose is useful in purification and identification of expressed VSV-G fusion proteins in bacterial lysates, or in transfected cells.

ChIP-seq

Data deposition

☒ Confirm that both raw and final processed data have been deposited in a public database such as GEO.

☒ Confirm that you have deposited or provided access to graph files (e.g. BED files) for the called peaks.

Data access links<br>*May remain private before publication.*	https://www.ncbi.nlm.nih.gov/bioproject/PRJNA748760
Files in database submission	Processed data files:<br>GcsR-V_AllPeaks.bed<br>GacA-V_AllPeaks.bed<br>FleQ-V_AllPeaks.bed<br>GcsR-V_A_ChIP_normalized.profile.wig.gz<br>GcsR-V_B_ChIP_normalized.profile.wig.gz<br>GcsR-V_C_ChIP_normalized.profile.wig.gz<br>GcsR-V_A_background_normalized.profile.wig.gz<br>GcsR-V_B_background_normalized.profile.wig.gz<br>GcsR-V_C_background_normalized.profile.wig.gz<br>GacA-V_A_ChIP_normalized.profile.wig.gz<br>GacA-V_B_ChIP_normalized.profile.wig.gz<br>GacA-V_C_ChIP_normalized.profile.wig.gz<br>GacA-V_A_background_normalized.profile.wig.gz<br>GacA-V_B_background_normalized.profile.wig.gz<br>GacA-V_C_background_normalized.profile.wig.gz<br>FleQ_A_ChIP_normalized.profile.wig.gz<br>FleQ_B_ChIP_normalized.profile.wig.gz<br>FleQ_C_ChIP_normalized.profile.wig.gz<br>FleQ_A_background_normalized.profile.wig.gz<br>FleQ_B_background_normalized.profile.wig.gz<br>FleQ_C_background_normalized.profile.wig.gz<br><br>Raw data files:<br>Mock_2_A_R1.fastq.gz<br>Mock_2_A_R2.fastq.gz<br>Mock_2_B_R1.fastq.gz<br>Mock_2_B_R2.fastq.gz<br>Mock_2_C_R1.fastq.gz<br>Mock_2_C_R2.fastq.gz<br>GcsR_A_R1.fastq.gz<br>GcsR_A_R2.fastq.gz<br>GcsR_B_R1.fastq.gz<br>GcsR_B_R2.fastq.gz<br>GcsR_C_R1.fastq.gz<br>GcsR_C_R2.fastq.gz<br>retS_Mock_A_R1.fastq.gz<br>retS_Mock_A_R2.fastq.gz<br>retS_Mock_B_R1.fastq.gz<br>retS_Mock_B_R2.fastq.gz<br>retS_Mock_C_R1.fastq.gz<br>retS_Mock_C_R2.fastq.gz<br>GacA_A_R1.fastq.gz<br>GacA_A_R2.fastq.gz<br>GacA_B_R1.fastq.gz<br>GacA_B_R2.fastq.gz<br>GacA_C_R1.fastq.gz<br>GacA_C_R2.fastq.gz<br>FleQ_A_R1.fastq.gz<br>FleQ_A_R2.fastq.gz<br>FleQ_B_R1.fastq.gz<br>FleQ_B_R2.fastq.gz<br>FleQ_C_R1.fastq.gz<br>FleQ_C_R2.fastq.gz
Genome browser session<br>(e.g. UCSC)	No longer applicable.

Methodology

Replicates	Experiments were performed with biological triplicate samples.
Sequencing depth	Between approximately 15–20 million paired-end 75 base pair reads were obtained for each biological replicate. Of these, 20–35% of

Sequencing depth

the reads mapped to the reference PAO1 genome, NC_002516.2, following in vitro size selection for fragments of less than 200 base pairs. This corresponds to 25 – 40x coverage of the reference genome.

Antibodies

Anti-VSV-G agarose beads (Sigma; part number A1970-1ML).

Peak calling parameters

Reads were mapped to the PAO1 genome (NC_002516) using bowtie2 version 2.4.1 allowing up to one mismatch per seed. The program QuEST (version 2.424) was used to call peaks. Reads collected from the PAO1 replicate mock IPs were merged and served as the "background" for each biological replicate for the GcsR-V IP's. Reads collected from the PAO1 ΔretS replicate mock IPs were merged and served as the "background" for each biological replicate for the GacA-V IP's. Peaks were called using the following parameters: KDE bandwidth = 30, Region = 200, Mappable genome fraction = 1, ChIP_enrichment_threshold = 1, ChIP_extension_enrichment = 1.5, ChIP_to_background_ratio = 2.

Data quality

Regions in each biological replicate were considered peaks if they are 3-fold enriched for reads over background, have a positive peak shift and strand correlation, and have a q-value of less than 0.01. GcsR peaks are defined as the minimal region identified in at least two biological replicates, resulting in 7 peaks ranging in enrichment from 92 – 3.2-fold enriched over background and 4 peaks with greater than 5-fold enrichment. GacA peaks are defined as the minimal region identified in at least two biological replicates, resulting in 3 peaks ranging in enrichment from 220 – 3.6-fold enriched over background and 2 peaks with greater than 5-fold enrichment.

Software

The program QuEST (version 2.424) was used to analyze ChIP-Seq data.
